# The RNA–Methyltransferase Misu (NSun2) Poises Epidermal Stem Cells to Differentiate

**DOI:** 10.1371/journal.pgen.1002403

**Published:** 2011-12-01

**Authors:** Sandra Blanco, Agata Kurowski, Jennifer Nichols, Fiona M. Watt, Salvador Aznar Benitah, Michaela Frye

**Affiliations:** 1 Wellcome Trust Centre for Stem Cell Research, University of Cambridge, Cambridge, United Kingdom; 2 CR-UK Cambridge Research Institute, Li Ka Shing Centre, Cambridge, United Kingdom; 3 Center for Genomic Research (CRG) and UPF, Institució Catalana de Recerca i Estudis Avançats (ICREA), Barcelona, Spain; Stanford University, United States of America

## Abstract

Homeostasis of most adult tissues is maintained by balancing stem cell self-renewal and differentiation, but whether post-transcriptional mechanisms can regulate this process is unknown. Here, we identify that an RNA methyltransferase (Misu/Nsun2) is required to balance stem cell self-renewal and differentiation in skin. In the epidermis, this methyltransferase is found in a defined sub-population of hair follicle stem cells poised to undergo lineage commitment, and its depletion results in enhanced quiescence and aberrant stem cell differentiation. Our results reveal that post-transcriptional RNA methylation can play a previously unappreciated role in controlling stem cell fate.

## Author Summary

We demonstrate that the RNA methyltransferase activity of Misu/NSun2 is required for the proper maintenance of the epidermal differentiation program, and thus post-transcriptional mechanisms are involved in controlling the balance between stem cell self-renewal and differentiation.

## Introduction

Stem cells are defined by their ability to continuously maintain their population (self-renewal) while generating progeny (differentiation). During self-renewal, stem cells have to avoid cell cycle exit and differentiation; however, when differentiating they have to evade uncontrolled proliferation. Thus, the question of how the balance between self-renewal and commitment is regulated is highly relevant to a fundamental understanding of cell differentiation and cancer.

The hair follicle offers an excellent model to study stem cell fate, as it undergoes cyclic bouts of growth (anagen), apoptosis-mediated regression (catagen) and rest (telogen) [1]. Multipotent hair follicle stem cells, located in a special microenvironment called the bulge, are slow cycling but exhibit long-term contribution to all hair compartments [2], [3]. At the early onset of hair growth, single bulge cells migrate out of their niche in telogen and undergo proliferation as progenitors before they differentiate into hair [4], [5].

Once a stem cell has left its niche, intrinsic and environmental cues converge to balance proliferation of progenitors with lineage-specific differentiation. For example, c-Myc is known to control the balance between stem cell expansion and differentiation [6], [7], [8]. When activated in epidermal stem cells, Myc triggers their exit from the stem cell compartment, induces proliferation of progenitors, and subsequently leads to lineage-specific differentiation [9], [10], [11].

Because the nucleolar protein Misu/NSun2 (*M*yc-*i*nduced *SU*N-domain-containing protein) is direct target gene of c-Myc [12], we considered the possibility that its RNA methyltransferase activity may represent a novel mechanism to regulate stem cell fate. Misu catalyzes the formation of 5-methylcytidine (m^5^C) in tRNA and possibly other RNA species [12], [13], [14]. Whereas the function of (cytosine-5) methylated DNA has been extensively analyzed [15], the functional roles of methylated RNA are largely unknown [16]. To date, Misu (NSun2) is one of only two identified m^5^C methylases with substrate specificity towards tRNA [13], [17], [18], [19].

Here, we demonstrate that Misu is required for normal tissue homeostasis in vivo. Expression of Misu is up-regulated in the hair follicle bulge at the entry of anagen. Deletion of Misu prolongs stem cell quiescence leading to a delay in initiation of anagen. Thus, our data reveals a post-transcriptional modification as a novel mechanism used by stem cells to precise and temporally accurate balance self-renewal and differentiation.

## Results

### Ablation of Misu blocks tRNA methylation and causes weight loss and partial alopecia

To functionally analyse the role of Misu *in vivo*, we generated a reporter-tagged loss-of-function mouse model using an ES cell line carrying a Gene Trap in intron 8 of the NSUN2 gene (GGTC-clone ID: D014D11). The methyltransferase activity of Misu is mediated by the conserved SUN domain encoded by exon 2 to 12 (Figure S1A; red box) [19]. Insertion of the Gene Trap and fusion to the reporter β-galactosidase leads to disruption of the catalytic domain of Misu (Figure S1A; blue box) [20]. We confirmed deletion of Misu and presence of β-galactosidase by gene-specific PCR (Figure S1B). Quantitative real-time PCR (QPCR) using a probe for NSun2 showed substantial reduction of full-length Misu in skin of heterozygous (+/−) and loss of expression in homozygous mice (−/−) (Figure S1C). Western Blot analysis demonstrated that Misu −/− mice lacked Misu protein (Figure S1D).

**Figure 1:**
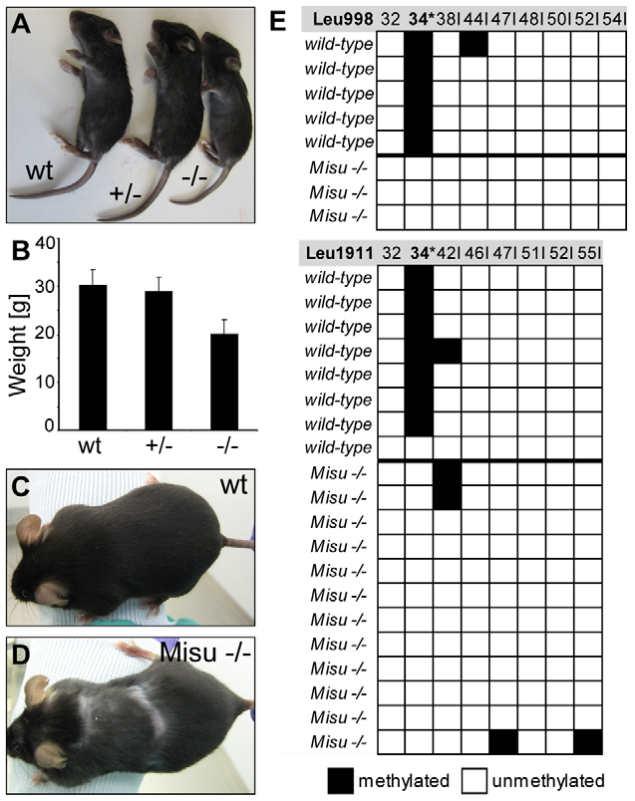
Loss of Misu results in block of tRNA methylation and causes reduced body weight and partial alopecia. (A) Gross phenotype of wild type, Misu +/− and −/− neonates. (B) Average weight of Misu −/− mice is reduced compared to controls. (C,D) Pelage of 10 months old wild-type (C) and Misu −/− mouse (D). Error bars represent standard deviation (SD). Wt: wild-type; +/−: heterozygous mice; −/−: homozygous mice. (E) Bisulfite sequencing of intron-containing tRNA^Leu (CAA)^ from wild-type and Misu −/− mouse skin. The diagram shows the methylation status of tRNA^Leu (CAA)^ transcribed from two different genes: tRNA-988^Leu (CAA)^ (top panel) and gene tRNA-1911^Leu (CAA)^ (bottom panel). Black boxes indicate methylated cytosine residues and white boxes indicate unmethylated cytosine residues. Numbers above boxes indicate the cytosine positions in the primary tRNA sequence amplified (nucleotides 29–36+intron sequence). Asterisk indicates the position of the expected methylated cytosine. Numbers+I indicate the cytosine positions in the intron sequence.

Heterozygous mice for Misu deletion were viable and did not display an obvious phenotype (Figure 1A). Homozygous mice were also viable, but were appreciably smaller than their wild type (wt) and heterozygous littermates (Figure 1A). At three months of age, Misu −/− mice weighed around 30% less than controls (Figure 1B). The smaller size of Misu −/− mice was confirmed by a second knock-out mouse model Nsun2^tm1a(EUCOMM)Wtsi^ generated by the Wellcome Trust Sanger Institute (http://www.sanger.ac.uk) (data not shown). Misu −/− males were sterile, and both genders had pelage that was comparable to controls after birth, but showed cyclic alopecia at around 10 months of age (Figure 1C, 1D).

Both, the yeast and human orthologue of Misu have previously been shown to catalyse the methylation of cytosine at position 34 (C34) in intron-containing pre-tRNA^Leu (CAA)^ [13], [19]. To investigate whether mouse Misu also methylated tRNA^Leu (CAA)^, we used RNA bisulfite sequencing, which allows analysis of RNA methylation patterns in their native sequence context [21]. We find that intron-containing pre-tRNA^Leu (CAA)^ was methylated at C34 in wild-type mice but lacked the modification when Misu was deleted (Figure 1E).

We concluded that Misu was indispensable for methylation of tRNA^Leu (CAA)^ in skin. Based on our observation that older mice show signs of alopecia in the absence of Misu we speculated that Misu might have a functional role in maintaining skin homeostasis in the long-term.

### Misu is dynamically expressed in embryogenesis and adult skin

**Figure 2:**
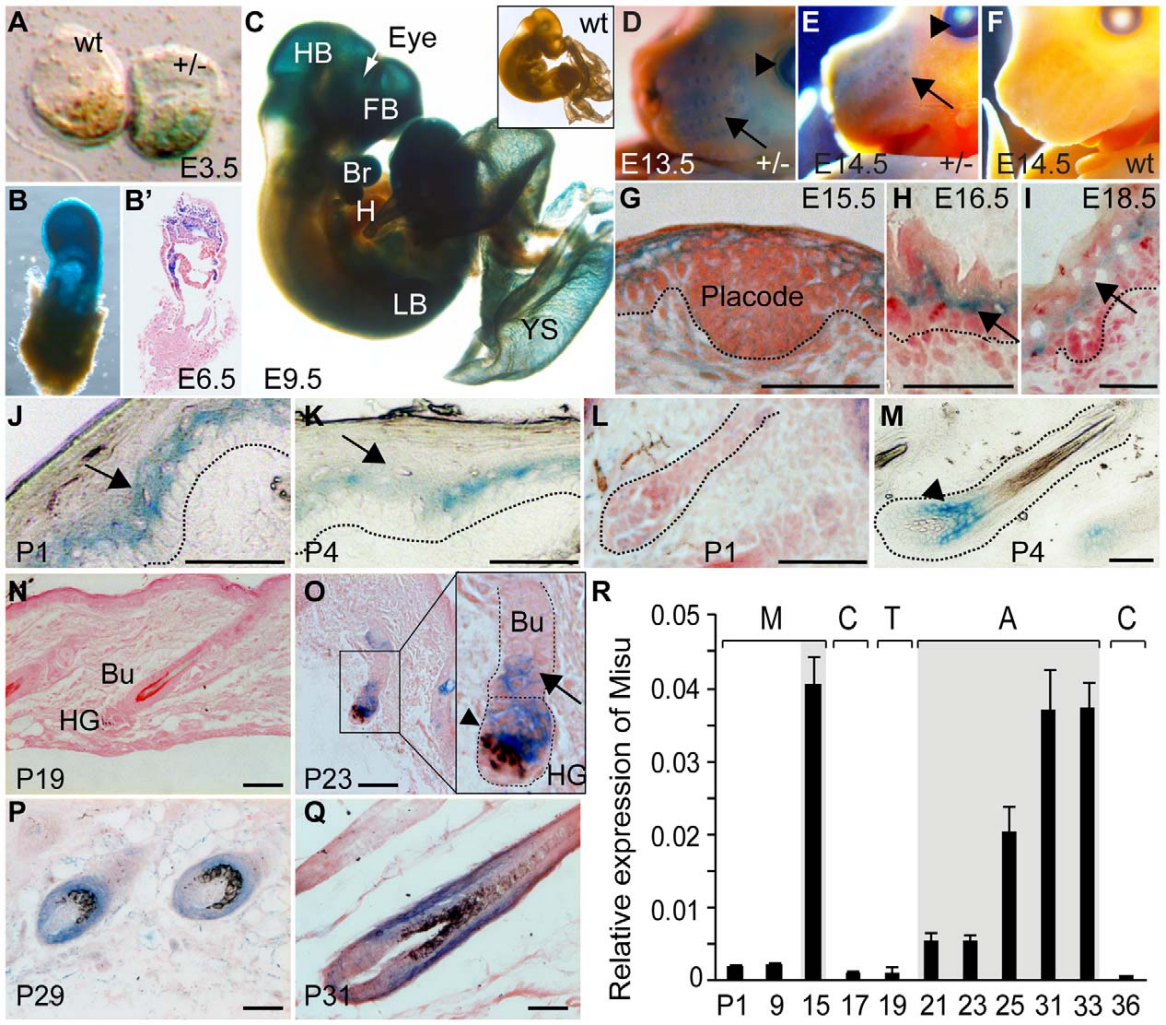
Expression of Misu during embryogenesis, skin morphogenesis, and in adult epidermis. (A–F) LacZ staining (blue) in wild-type (wt) and Misu (+/−) embryos at E3.5 (A), E6.5 (B,B′), E9.5 (C), E13.5 (D) and E14.5 (E,F). HB: hindbrain; FB: forebrain; Br: branchial arch; H: heart; LB: limbs; YS: yolk sac. (B′) shows a section from the embryo whole mount in (B). (D,E) Arrows show LacZ staining in whiskers. Arrowheads indicate Misu expression in the eye. (G–I) LacZ staining of Misu (+/−) interfollicular epidermis at E15.5 (G), E16.5 (H) and E18.5 (I). (J,K) Misu-expression in the interfollicular epidermis after birth at P1 (J), P4 (K). (L,M) LacZ staining of hair follicles at P1 (L) and P4 (M). Dotted lines delineate the basement membrane in (J,K) and hair follicles in (L,M). Arrows in (J,K) indicate suprabasal layers of the interfollicular epidermis. Arrowhead in (M) indicate Misu-expression in the bulb of growing hair follicles. (N–R) Expression of Misu in adult skin is absent in telogen (N), low expressed in the bulge (Bu; arrows) and hair germ (HG; arrowhead) at early anagen (O) and high expressed in anagen hair follicles (P,Q). (R) mRNA levels of endogenous Misu during morphogenesis (M), catagen (C), telogen (T) and anagen (A). The grey box indicates anagen. Error bars indicate SEM (n = 2). Sections are counterstained with Eosin in (G–I, L, N–Q). Scale bars: 50 µm (G–Q).

To determine the role of Misu in skin, we began by examining endogenous expression of Misu during embryogenesis and skin development by staining for LacZ (Figure 2A–2Q). Misu-expression was detected from E3.5 in the inner cell mass of the blastocyst (Figure 2A). After implantation and gastrulation, at E6.5, Misu was observed throughout the extra-embryonic ectoderm (Figure 2B, 2B′), which gives rise to the nervous system and epidermis. Starting from E9.5, expression of Misu became more restricted and at E13.5 and E14.5 Misu was enriched in developing whiskers (arrow) and eyes (arrowhead) (Figure 2C–2F). From E15.5, when the interfollicular epidermis (IFE) begins to stratify and follicular morphogenesis starts by forming hair placodes (Figure 2G), highest expression of Misu was found in the suprabasal layer of IFE (Figure 2G–2I; arrows).

After birth, from postnatal day 1 (P1), expression of Misu in the IFE waned (Figure 2J, 2K, 2N, arrows) but increased in the matrix of growing (anagen) hair follicles (Figure 2L, 2M, arrowhead). At the end of follicular morphogenesis, from around P14 to P19, when hair follicles progress through the destructive (catagen) and resting (telogen) phase of the hair cycle, expression of Misu was absent (data not shown and Figure 2N). At early anagen (P23), Misu was up-regulated in the hair germ (HG; arrowhead) and weakly expressed in the bulge (Bu; arrow) (Figure 2O; insert). Throughout anagen, Misu was highly expressed in matrix but was also found in differentiated lineages of the hair follicles (Figure 2P, 2Q, Figure S2). We confirmed the dynamic expression pattern of Misu in skin by detecting endogenous RNA levels of Misu during morphogenesis (M), catagen (C), telogen (T), and anagen (A) of the first postnatal hair cycle by QPCR (Figure 2R).

In conclusion, Misu was dynamically expressed during morphogenesis and in adult skin. In adult skin, expression of Misu was up-regulated in the bulge and hair germ as soon as the hair follicle entered its growing phase (anagen). During anagen, cells of the hair germ give rise to the hair matrix, which showed highest expression of Misu. Matrix cells, which are often referred as to transit amplifying (TA) cells because they only survive through anagen [22], are stem cell progenitors that divide a finite number of times until they become differentiated.

### Misu is a transcriptional target of the Lef1/β-Catenin complex in hair follicles

**Figure 3:**
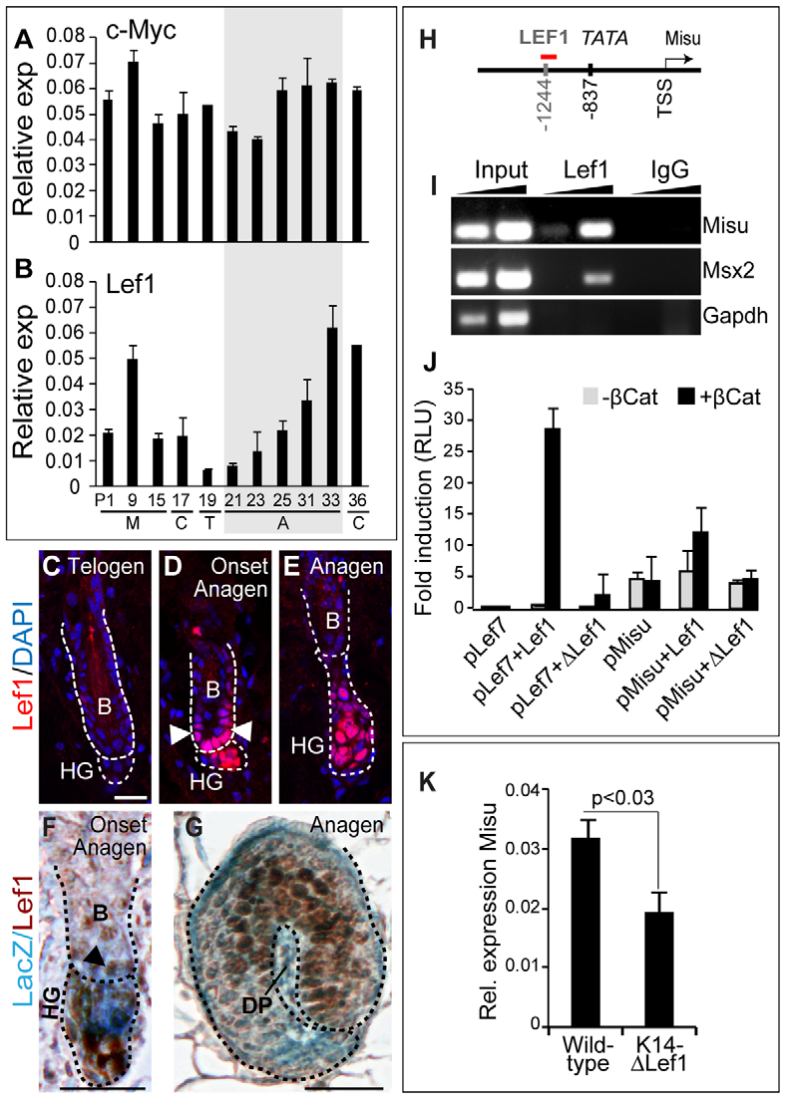
Misu is a down-stream target gene of the Lef1/β-Catenin complex in adult skin. (A–B) QPCR for c-Myc (A) and Lef1 (B) using total RNA isolated from back skin of wild-type mice during hair morphogenesis and the first hair cycle after birth. M; morphogenesis, C; catagen, T; telogen and A; anagen. The grey box marks anagen. Error bars represent SEM (n = 3). (C–E) Localisation of Lef1 protein (red) in the bulge (B, arrowheads) and hair germ (HG) at telogen (C), onset of anagen (D) and anagen (E). (F,G) Co-localisation of Misu (LacZ) and Lef1 (brown) in the lower bulge (B, arrowheads) and hair germ (HG) at the onset of anagen (F) and in the matrix later in anagen (G). DP indicates the dermal papilla. (H) Schematic overview of the Misu promoter indicating the putative Lef1-binding site, the TATA box and the transcriptional start site (TSS) for Misu. The red line indicates the PCR fragment amplified after ChIP. (I) Enrichment of promoters of Misu and as positive control Msx2 by ChIP using an antibody for Lef1. IgG and Gapdh served as negative controls. (J) Luciferase reporter assays using 2 kb of the mouse proximal promoter of Misu in Hela cells in the absence and presence of β-Catenin, Lef1 or ΔN63-Lef1. pLef7-fos-luc synthetic promoter was used as a positive control for Lef1/β-Catenin activity. Error bars represent SD (n = 3). (K) RNA levels of Misu in wild-type and K14-ΔLef1 skin. Nuclei are counterstained with DAPI (blue) (C–E). Scale bars: 20 µm (C–E). Scale bar: 25 µm (F,G).

Misu was originally identified as a transcriptional target of c-Myc in skin [12]. However, activation of c-Myc triggers epidermal stem cells to differentiate mainly into lineages of IFE and sebaceous glands [7]. Since a role for c-Myc in regulating bulge stem cells has yet not been identified and c-Myc expression levels remained unchanged during the hair cycle (Figure 3A), we asked whether expression of Misu might also be regulated by hair-specific transcription factors. We examined the mouse Misu proximal promoter and detected a putative Lef1-binding motif (Figure 3H). As described for Misu, also expression of Lef1 increased when hair follicles entered anagen (Figure 2R; Figure 3B, 3C) and expression of Misu and Lef1 overlapped in hair follicles at both early and late stages of anagen (Figure 3F, 3G).

To validate Misu as a target gene of Lef1, we performed chromatin immunoprecipitation (ChIP) in mouse epidermis in anagen using an antibody for Lef1 (Figure 3I). We detected Lef1-binding to the promoters of Misu and Msx2, a known target gene of Lef1 [23]. We further confirmed Misu as a transcriptional target of the Lef1/β-catenin complex by luciferase assays using the Misu promoter (pMisu) and full-length Lef1 or ΔN63Lef1 (ΔLef1), a mutant construct lacking the β-catenin binding motif. The assays were performed in presence or as an additional negative control absence of β-Catenin (Figure 3J). The Lef7 synthetic promoter (pLef7) served as positive control (Figure 3J). Luciferase activity using the Misu promoter increased around two-fold when the Lef1/β-catenin complex was present compared to the controls (Figure 3J). Finally, we confirmed *in vivo* that Misu RNA levels decreased when ΔLef1 was over-expressed in the mouse epidermis (K14ΔLef1) (Figure 3K). We concluded that expression of Misu is up-regulated by Lef1 when hair follicles enter anagen.

### Misu is expressed in bulge stem cells and the hair germ at the initiation of anagen

**Figure 4:**
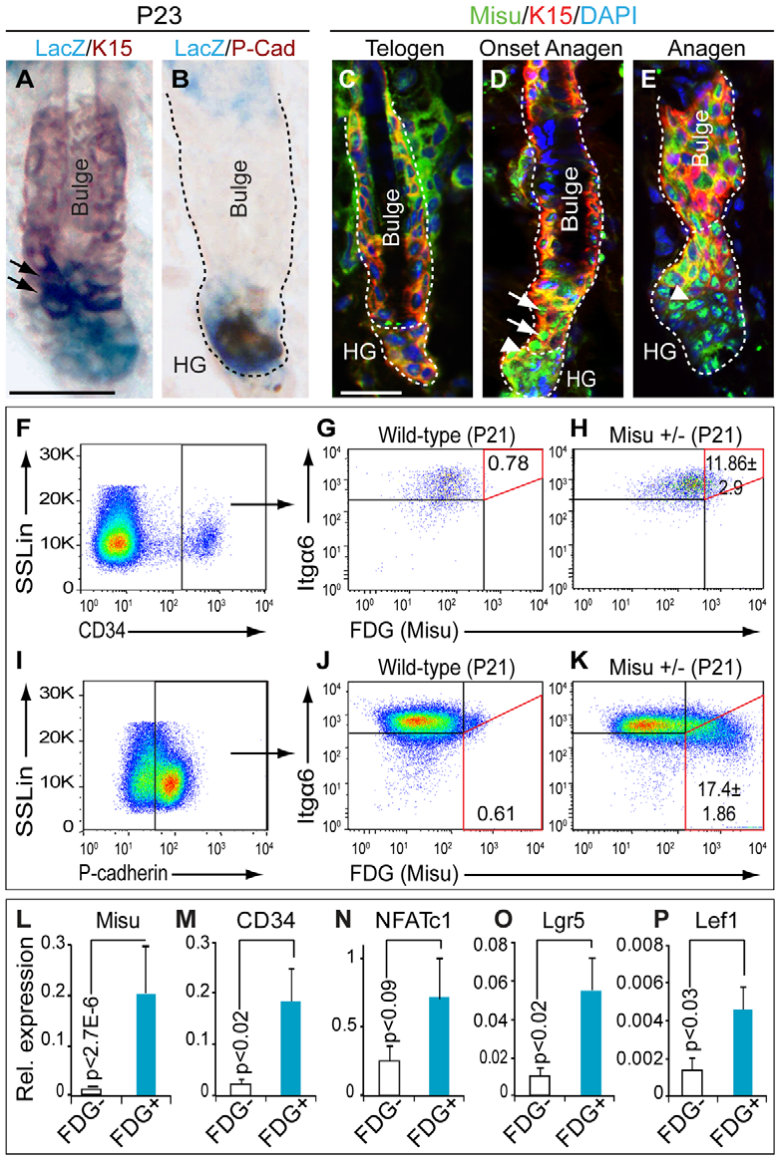
Misu is expressed in bulge stem cells. (A,B) Co-localisation of LacZ and keratin 15 (K15) (A) and P-cadherin (PCad) (B) in Misu +/− hair follicles. Arrows in (A) indicate K15/LacZ double positive cells. (C–E) Expression of Misu protein (green) in bulge (arrows) and hair germ (HG; arrowheads) at telogen (C), onset of anagen (D) and anagen (E). Bulge cells are labelled for K15 (red) and nuclei are counter stained with DAPI (blue) (C–E). (F–K) Percentage of FDG^+ve^ sorted epidermal cells in the bulge (Itgα6^high^/CD34^+ve^) (F–H) and the hair germ (Itgα6^low^/P-cadherin^high^) (I–K) at P21 in Misu +/− mice. (F,I) Cells were first gated by side scatter (SSLin) versus high expression of CD34 (F) or P-cadherin (I). The cells were further gated for Itgα6^high^/FDG^+ve^ (G,H) and Itgα6^low^/FDG^+ve^ (J,K). The percentage of Itgα6^high^/CD34^+ve^/FDG^+ve^ and Itgα6^low^/P-cadherin^high^/FDG^+ve^ cells is marked by the red squares (G,H,J,K). Wild-type cells were negative for FDG (G,J). Errors represent SEM (n = 3). (L–P) QPCR using FDG^+ve^ and FDG^−ve^ epidermal cells sorted from Misu +/− mice at P21 as indicated in Figure S6 for Misu (L), CD34 (M), NFATc1 (N), Lgr5 (O), and Lef1 (P). Error bars represent SEM (n = 3). Scale bars: 50 µm (A–E).

The complete lack of expression of Misu in adult skin in both the IFE and the bulge in either the catagen or telogen phase of the hair cycle excludes its expression in quiescent stem cells. However, Misu-expression, detected by LacZ staining, was up-regulated in the bulge region (arrows) and the hair germ (HG) as early as telogen to anagen transition (Figure 4A, 4B; Figure S3A, S3B). We confirmed expression of Misu protein in the bulge (arrows) and the hair germ (arrowheads) in anagen by immunoflourescence using an antibody against mouse Misu (Figure 4C–4E; Figure S3C).

We next asked whether Misu-positive cells in the bulge were indeed stem cells. Hair follicle stem cells can be isolated by fluorescence activated cell sorting (FACS), based on high expression of CD34 and α6 integrin (Itgα6) (Figure 4F–4H) [24]. Progenitor cells of the hair germ are characterized by high expression of P-cadherin and low expression of Itgα6 (Figure 4I–4K; Figure S4A–S4C) [5]. Both, stem and progenitor populations were sorted from Misu +/− mice at the onset of anagen (P21) and tested for expression of Misu based on β-galactosidase activity (LacZ) using fluorescein di-galactoside (FDG) (Figure 4F–4K). Around 12% of bulge stem cells (Itgα6^high^/CD34^+ve^) and 17% of progenitor cells in the hair germ (Itgα6^low^/P-cadherin^high^) expressed Misu (Figure 4F–4K). No signal for FDG was detected in keratinocytes from wild-type mice (Figure 4G, 4J).

To further confirm co-expression of Misu with stem cells markers, we isolated FDG^+ve^ and FDG^−ve^ keratinocytes from Misu +/− mice at P21 (Figure S5A, S5B). QPCR analysis demonstrated that the stem cell markers CD34, NFATc1, and Lgr5 were enriched in Misu-expressing cells (FDG^+ve^) (Figure 4L–4O). Both populations expressed Itgα6 at similar levels (Figure S5C) but FDG^+ve^ cells were also enriched for the hair germ markers Lef1, Wnt5a, and Sox6 (Figure 4P; Figure S5D, S5E). Expression of differentiation markers was comparable or decreased compared to FDG^−ve^ cells (Figure S5F, S5G). We concluded that Misu was expressed in both bulge stem cells and cells of the hair germ at initiation of anagen.

### Lack of Misu delays cell cycle entry of bulge stem cells at telogen–anagen transition

**Figure 5:**
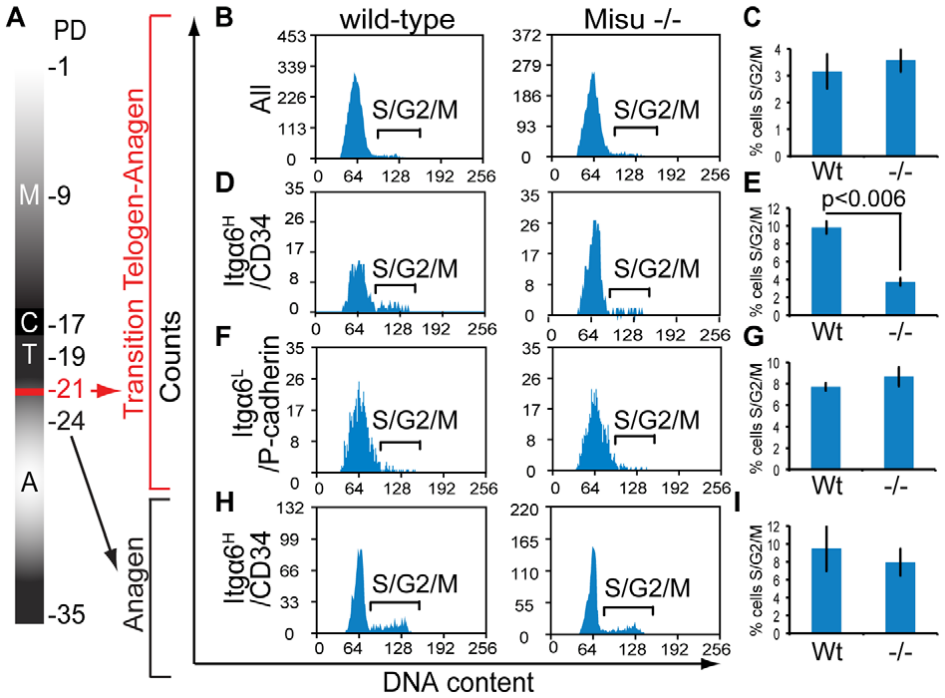
Misu delays cell cycle entry of bulge stem cells at the onset of anagen. (A) Schematic representation of the first postnatal hair cycle after morphogenesis (M) including catagen (C), telogen (T) and anagen (A). Postnatal days (PD) are indicated on the right. Red marks the transition of telogen to anagen at P21. (B–I) Cell cycle profiles (B,D,F,H) and quantification (C,E,G,I) of whole epidermal preparations (B,C), Itgα6^high^/CD34^+ve^ bulge stem cells (D,E), Itgα6^low^/P-cadherin^high^ hair germ cells (F,G) and Itgα6^high^/CD34^+ve^ bulge stem cells at P24 (H,I) using a minimum of three wild-type and three Misu −/− mice. Error bars represent SEM.

To test whether Misu might induce bulge stem cells to enter cell cycle at the transition of telogen to anagen, we FACS-sorted bulge stem cells, based on high expression of CD34 and Itgα6 (Figure S6A) and early progenitor cells of the hair germ, based on high expression of P-cadherin and low expression of Itgα6 (Figure S6B) at the onset of anagen at P21 in wild-type and Misu −/− mice. We then determined the cell cycle profile for all sorted populations (Figure 5A–5I). We did not observe any difference in cell cycle profile when the whole epidermal population was analyzed, and as expected most of the cells were in G1 (Figure 5B, 5C). However, the percentage of Misu −/− bulge stem cells (Itgα6^high^/CD34^+ve^) in S- and G2/M-phase of the cell cycle was significantly reduced compared to wild-type cells at anagen initiation (Figure 5D, 5E). In contrast, the cell cycle profile of progenitor cells (Itgα6^low^/P-cadherin^high^) in Misu −/− and wild-type epidermis was comparable (Figure 5F, 5G). At later stages, in anagen at P24, the percentage of Misu −/− bulge stem cell population dividing was comparable to that of wild-type controls (Figure 5H, 5I; Figure S6C). These data indicated that Misu was important to stimulate cell cycle entry of bulge stem cells at initiation of anagen and depletion of Misu might increase the quiescent phase of bulge stem cells.

### Deletion of Misu extends quiescence of bulge stem cells

**Figure 6:**
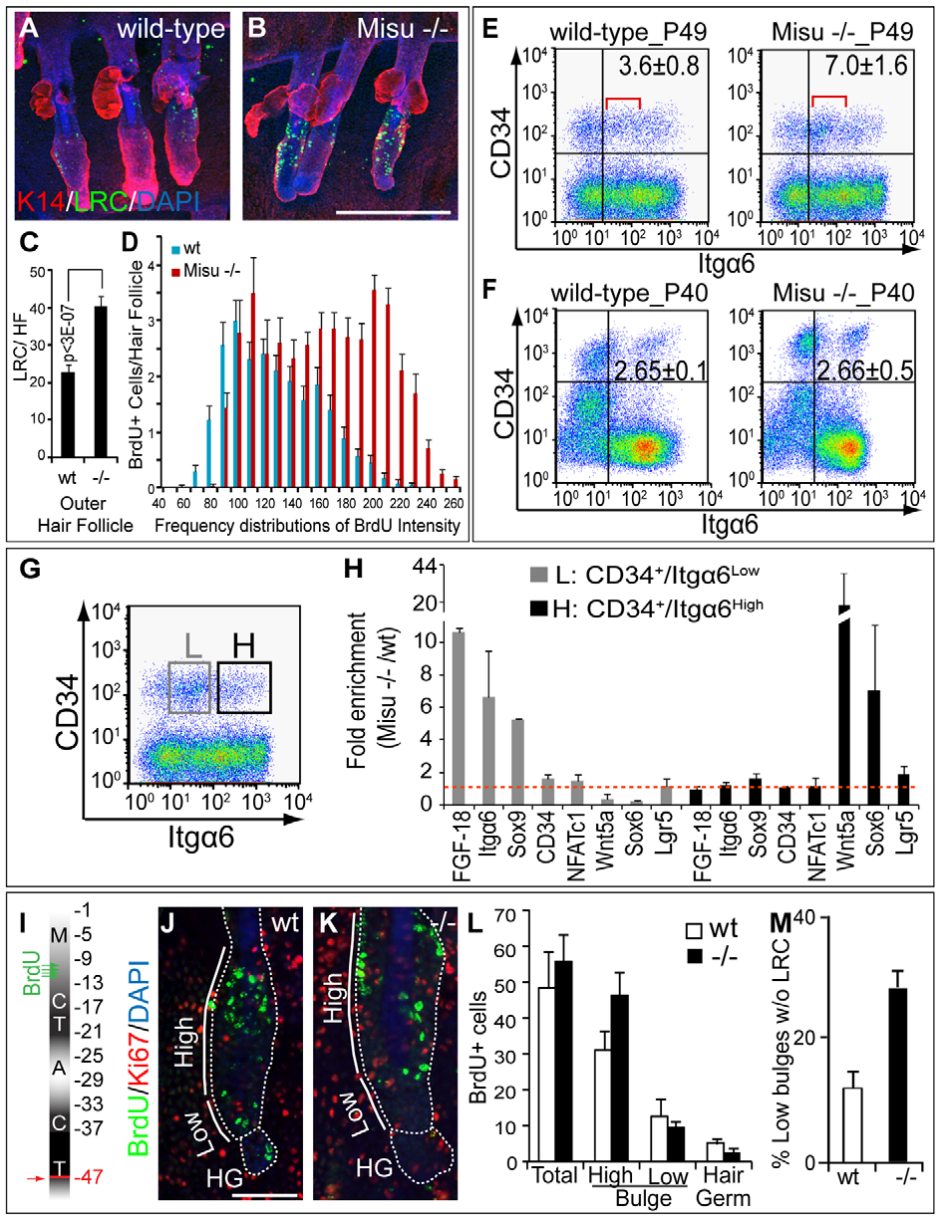
Deletion of Misu increases dormancy of bulge stem cell. (A,B) Whole mount staining of wild-type and Misu −/− tail skin for LRC (green), keratin 14 (K14) (red) and DAPI (blue) after 4 months chase period. (C) Automated quantification of LRC in tail outer hair follicles in (A,B). (D) Frequency distributions of the intensity of the BrdU-label in LRC in (A,B). Error bars represent SEM (n = 10) from at least 3 independent experiments. (E,F) Flow cytometry for Itgα6 and CD34 in epidermis in telogen (P49) (E) and catagen (P40) (F). The percentage of double positive cells in each group is shown ± SEM (n = 3). The red lines in (E) indicate a cell population that increases in Misu −/− skin in telogen. (G,H) Epidermal cells sorted for CD34^+ve^ and low (L) and high (H) levels of Itgα6 were subjected to QPCR for bulge and hair germ markers as indicated (G). RNA levels were measured relative to GAPDH and presented as fold enrichment in Misu −/− mice versus wild-type controls. Error bars represent SEM (n = 3). (I) BrdU labelling and chasing regime to measure migration of LCR from bulge to hair germ at telogen at P47 (arrow). (J,K) Detection of LRC (green), Ki67 (red) and DAPI (blue) in high and low part of the bulge (straight line) and hair germ (HG) in hair follicles, indicated by dotted line, from wild-type (wt) (J) and Misu −/− mice (K). (L) Automated quantification of LRC in the whole hair follicle (total), high and low bulge region and the hair germ. Error bars indicate SEM (n = 5). (M) Percentages of hair follicles without LRC in the lower bulge region. Scale bars: 250 µm (A,B); 50 µm (J,K).

To test our hypothesis that lack of Misu resulted in increased number of quiescent bulge stem cells, we labelled Misu −/− and wild-type mice with BrdU, and detected label-retaining cells (LRC) after a chase period of four months (Figure 6A–6D). A long chase period allows detecting differences not only in the number of LRC but also in the intensity of the BrdU-label, which correlates with number of divisions. The number of LRC in Misu-depleted tail hair follicles was significantly increased in the outer follicles of triplets (Figure 6A–6C), which are known to go through the hair cycle concurrently, whereas the central follicle cycles asynchronously and usually has fewer LRC (data not shown) [25], [26], [27]. Additionally, the intensity of the BrdU-label was higher in Misu −/− skin suggesting that those cells divided slower than in their wt controls (Figure 6D).

Flow cytometry for CD34 and Itgα6 in anagen (P30), catagen (P40) and telogen (P49) confirmed that loss of Misu resulted in a two-fold increase of bulge stem cells only in telogen of the hair cycle (Figure 6E, 6F; Figure S7A). The number of hair germ cells in telogen (P49) was unchanged (Figure S7B). Strikingly, we found that the increase of bulge stem cells in Misu −/− epidermis was due to an enrichment of a distinct cell population with lower Itgα6 expression compared to wild-type skin (Figure 6E; red line), which might represent those bulge cells that failed to enter the cell cycle at the initiation of anagen (Figure 5D). If our hypothesis was correct CD34^+ve^ but Itgα6^low^ cells from Misu −/− mice should be more quiescent and therefore express higher levels of bulge stem cell markers than the comparable wild-type population.

To test our hypothesis, we sorted bulge stem cells into two populations: L (CD34^+ve^/Itgα6^low^) and H (CD34^+ve^/Itgα6^high^) (Figure 6G). We then analysed both populations for expression of bulge markers in wild-type and Misu −/− epidermis (Figure 6H). We found that population L obtained from Misu −/− mice showed consistently higher expression of the stem cell markers FGF-18, Itgα6, Sox9, CD34 and NFATc1 relative to wild-type mice (Figure 6H; Figure S8E). Importantly, population L did not show increased expression of the hair germ markers Wnt5a and Sox6, or Lgr5, a marker for cycling stem cells (Figure 6H). In contrast, population H from Misu −/− mice expressed similar levels of stem cell markers than wild-type mice, but showed an increase in the expression of hair germ markers. Thus, our data indicated that depletion of Misu resulted in an accumulation of quiescent CD34^+ve^/Itgα6^low^ stem cells in the bulge.

To exclude the possibility that the increase in the quiescent stem cell population in Misu −/− mice was due to the general lack of Misu rather than being tissue-autonomous to skin, we generated a conditional knockout mouse model for Misu in the epidermis (Methods) (Figure S8A–S8C). Like in Misu −/− animals, also in mice with conditionally deleted Misu in the basal, undifferentiated layers of the epidermis (K14Misu^Δ/Δ^), a distinct cell population of stem cells with lower Itgα6 expression accumulated in the bulge (Figure S8D; red line in right hand panel) and expressed higher levels of stem cell markers than the Cre-negative Misu^f/f^ controls (Figure S8E).

### Deletion of Misu delays exit of stem cells from the bulge

*In vivo* lineage tracing experiments suggest that a sub-population of bulge cells migrate into the hair germ in telogen to then undergo cell division at the onset of anagen [4], [28]. We speculated that stem cells in the bulge of Misu −/− mice might fail to commit in the bulge and do not migrate into the hair germ. To determine whether lack of Misu affected stem cell migration into the hair germ, we labelled Misu −/− new born mice and control littermates with BrdU at late morphogenesis and chased them for one hair cycle until the second postnatal telogen (P47) We then detected quiescent bulge stem cells using an antibody to BrdU and compared the number and location of label-retaining cells (LRC) in the upper (high) and lower bulge region and the hair germ (Figure 6I–6M; Figure S9A–S9F) [4].

Wild-type and Misu −/− epidermal cells incorporated BrdU at the same rates (Figure S9A, S9B). After the chase period at P47, we found a significant higher number of fully labelled LRC in the high bulge area of Misu −/− epidermis (Figure 6J–6L). Moreover, the number of hair follicles without any LRC in the lower bulge or hair germ was three-fold higher in Misu −/− skin than wild-type littermates (Figure 6M). Accordingly, we also found that the intensity of the BrdU-label increased in the higher bulge and decreased in the lower bulge and hair germ when Misu was depleted (Figure S9C–S9F). Importantly, at this stage of the hair cycle the majority of bulge cells are yet not dividing, as shown by labelling for Ki67 (Figure 6J, 6K), excluding the possibility that Misu −/− cells diluted the label by cell divisions.

Our data showed that the increased quiescence of bulge stem cells in Misu −/− skin correlated with an accumulation of LRC in the upper part of the bulge, indicating that Misu −/− bulge stem cells are delayed in generating committed progenitor cells of the hair germ.

### Deletion of Misu increases stem cell self-renewal and causes aberrant hair cycling

**Figure 7:**
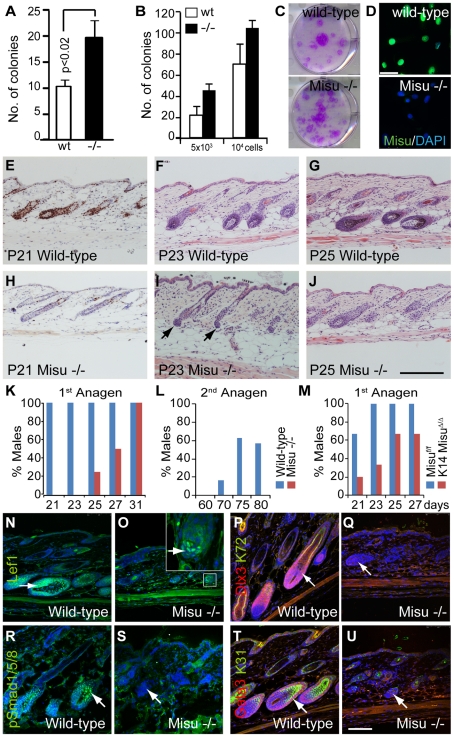
Lack of Misu affects self-renewal of epidermal stem cells. (A,B) Colony forming efficiency of flow-sorted bulge stem cells (A) and 5×10^3^ or 10^4^ whole epidermal cells isolated from wild-type (wt) and Misu −/− back skin (B). Error bars represent SD (n = 3). (C) Representative culture dishes from (B). (D) Confocal images of wild-type and Misu −/− primary keratinocytes in culture stained for Misu (green) and DAPI (blue). (E–J) Ki67 staining (E,H) and Haematoxylin and Eosin staining (F,G,I,J) of epidermal sections taken at them same regions of dorsal skin at indicated postnatal days obtained from anagen in Misu −/− and wild-type mice. (K–M) Percentages of male wild-type and Misu-depleted males in the first adult anagen (K), the second adult anagen (L), and the first anagen in K14Misu^Δ/Δ^ and Misu^f/f^ mice (M) at time points indicated. Detailed numbers of animals are summarized in Table S1. (N–U) Delayed expression of signalling molecules in growing hair follicles of Misu −/− mice compared to wild-type littermates at P25: Lef1 (green) (N,O), hair keratin 72 (green) and Dlx3 (red) (P,Q), phospho-Smad1/3/5 (green) (R,S) and hair keratin 31 (green) and Gata3 (red) (T,U). Nuclei are counter stained with DAPI (blue). Insert in (O) shows higher magnification of matrix cells. Arrows in (I) mark dermal papilla and arrows in (N–U) indicate matrix cells. Scale bars: 50 µm (D), 200 µm (E–J) and 100 µm (N–U).

To test whether Misu-deletion led to increased self-renewal capacity of stem cells *in vitro*, we measured the colony forming efficiency (CFE) of sorted Itgα6^high^/CD34^+ve^ bulge stem cells (Figure 7A). Out of all cell populations derived from skin, epidermal stem cells exhibit the highest CFE [29]. When seeded in clonal density, keratinocytes derived from Misu −/− mice formed more colonies than wild-type littermates (Figure 7A), indicating that Misu −/− cells have a higher self-renewal capacity than wild-type cells. Similarly, unsorted keratinocytes obtained from Misu −/− epidermis showed higher CFE than control keratinocytes (Figure 7B, 7C). Although expression of Misu was undetectable in adult IFE (Figure 2N), cultured keratinocytes obtained from back skin expressed high levels of Misu RNA and protein (Figure 7D; data not shown).

Our data indicated so far, that loss of Misu increased accumulation of bulge stem cells in their niche at late telogen leading to an increased self-renewing but quiescent stem cell population. Therfore, we speculated that Misu −/− hair follicles should be delayed in entering anagen. Indeed, depletion of Misu led to a delay of entry into the first and second synchronized hair cycle in males (Figure 7E–7L; Figure S10B, S10C). Compared to males, females exhibit a delayed hair cycle progression of around 2 days, yet even in Misu −/− females the percentage of hair follicles in the first anagen at P25 was lower than in their wild-type controls (Figure S10A; Table S1). Similarly, male mice with conditionally deleted Misu in the epidermis (K14Misu^Δ/Δ^), displayed a delay in entering the first adult hair cycle compared to controls (Misu^f/f^) (Figure 7M; Figure S10F–S10K).

Later entry into anagen in Misu-depleted skin resulted in delayed differentiation of matrix cells (Figure 7N–7U). The number of Lef1-postive cells, marking lineage committed hair follicle cells, was lower in matrix cells of Misu −/− mice (Figure 7N, 7O; arrows) [30]. Accordingly, expression of Dlx3, Gata3 and BMP signalling, as determined by staining for phosphorylated Smad1/5/8, were absent in Misu −/− anagen hair follicles (Figure 7P–7U; arrows). Once Misu −/− mice entered anagen, the hair follicles were morphologically indistinguishable from those of wild-type mice (Figure S10F, S10E, S10L–S10S). Our data suggested that Misu plays a role in accurately timing lineage commitment of hair follicle progenitor cells.

## Discussion

### The RNA methyltransferase Misu balances self-renewal and differentiation

Here we show through generating general and skin-specific loss-of-function mouse models that the RNA methyltransferase Misu (NSun2) defines expanding, committed progenitor populations in mammalian skin. Expression of Misu is absent in adult mouse interfollicular epidermis and the quiescent phases of the hair cycle (catagen and telogen). As the hair follicle enters its growing phase (anagen), Misu is expressed in the bulge and hair germ, both of which contain multipotent stem cells [3], [31], [32]. Cells in the hair germ give rise to transit amplifying (TA) cells in the hair matrix [4], [5]. Matrix cells, which collectively show the highest expression of Misu of all cell types, subsequently differentiate into all hair lineages [3].

Uniquely at the transition of telogen to anagen, Misu is co-expressed with markers for both quiescent (CD34^+ve^/Itgα6^high^) and cycling (Lgr5^+ve^) stem cells from the bulge and the hair germ respectively. However, in contrast to those stem cell populations, Misu- expressing cells exhibit reduced self-renewal capacity. Thus, although Misu could reversibly commit stem cells to differentiate [33], we clearly show that stem and committed progenitor cells co-exist within the bulge, the hair follicle stem cell niche. Our findings now raise the question of how only a few selected stem cells are activated during each hair cycle.

Misu is required for cellular division of bulge cells only at the telogen-to-anagen transition, indicating that its function is temporarily and spatially controlled in a strict manner. One key pathway that drives epidermal stem cells from telogen into anagen and specifies hair follicle lineages is the canonical Wnt pathway. Activation of Wnt signaling by transient expression of N-terminally truncated β-catenin in the epidermis is sufficient to induce ectopic hair follicle formation [34], [35], [36], [37], [38]. Conversely, when the pathway is inhibited by β-catenin ablation or expressing of a ΔNLef1 mutant, hair follicle formation is impaired [30], [39], [40], [41]. The identification of Misu as a direct downstream target of Lef1 further supports Misu as a key component during lineage commitment of bulge stem cells at the initiation of anagen. Thus, our data support a model in which stem and committed progenitors are distinct populations within the hair follicle that can be distinguished by their expression of Misu.

### m^5^C RNA methylation and commitment to differentiate

Misu belongs to a large family of highly conserved methyltransferases, modifying cytosine-5 in RNA (RNA:m^5^C-MTase) [16]. Misu/NSun2 is the human orthologue of *S. cerevisiae* Trm4, both of which have substrate specificity towards tRNA [12], [13], [19]. Like for human and yeast NSun2, we confirm pre-tRNA^Leu (CAA)^ as a direct target substrate for mouse Misu *in vivo*. Although post-transcriptional methylation of tRNA at cytosine-5 is one of the most frequently encountered modifications, Dnmt2 and Misu are as yet the only identified tRNA:m^5^C MTases [13], [17], [18], [42]. Although it has been recently shown that m^5^C methylation protects tRNA from cleavage and degradation, the biological function this may mediate remains unclear [17], [43].

Similar to the depletion of Misu in mice, loss of Dnmt2 in zebrafish also results in reduced body size and impaired differentiation of specific tissues [44]. It remains, however, unclear why the RNA-methyltransferase activity of both proteins is critical in maintaining tissue homeostasis [44]. One possible mechanism of Misu's function is that methylated tRNA species could be directly involved in regulating stem cell differentiation. An alternative and intriguing possibility could be that methylation regulates the cleavage of tRNAs or intron-splincing of pre-tRNAs to generate products with microRNA-like features, which may offer an additional mechanism of post-transcriptional control [17], [45]. Recent RNA deep-sequencing data identified a new set of small RNAs derived from tRNAs, including intron sequences, which are associated with Dicer and Argonaute proteins, strongly suggesting a role of these fragments in RNA silencing [46], [47].

On the other hand, modified nucleotides in tRNA are well known to affect their structural and metabolic stability, and thus are likely to influence directly the rate or efficiency of protein translation [42], [48]. Interestingly, protein translation is hierarchically controlled during stem cell self-renewal and differentiation; while parsimonious during self-renewal, it enhances during differentiation [49]. This increased protein synthesis capacity in stem cells could allow rapid elevation of translational rate in response to differentiation signals [49].

In summary, we demonstrate that the RNA methyltransferase Misu (Nsun2) stimulates a sub-population of stem cells to leave the hair bulge and become committed progenitor cells in the hair germ. Thus we identify post-transcriptional RNA modifications as a novel mechanism by which stem cells control the balance between stem cell self-renewal and differentiation.

## Materials and Methods

### Ethics statement

All mouse husbandry and experiments were carried out according to the local ethics committee under the terms of a UK Home Office license.

### Mice

Misu −/− mice were derived using 129S2 ES cell line carrying a Gene Trap in intron 8 of the NSUN2 gene (GGTC-clone ID: D014D11) generated by the German Gene Trap Consortium, and then mated with C57Bl6/J CBA F1 mice. F1 progeny was subsequently inbred. A PCR-based strategy was developed to distinguish the wild-type and Misu gene trap alleles. The primers are as follows: SR2, (5′GCC AAA CCT ACA GGT GGG GTC TTT) and B34 (5′- TGT AAA ACG ACG GGA TCC GCC) amplify a fragment 650 bp of the ß-geo cassette. The primers Misu-Int8-5′ (5′AGG TGG ACC TGA TCA TGG AG) and Misu-Int8-3′ (5′-AGGG AGG GTC TGG AAA GATG) amplify a fragment of 500 bp of the wild-type allele.

Mice containing a floxed allele of the NSUN2 gene were obtained by first crossing Nsun2^tm1a(EUCOMM)Wtsi^ mice, generated by the Wellcome Trust Sanger Institute, with transgenic mice expressing Flp recombinase to delete the LacZ-neo cassette [50]. The offspring, containing two LoxP sites flanking exon 6, were then crossed with KRT14-cre mice (The Jackson Laboratory). In KRT14-cre mice, Cre-recombinase is expressed under the control of the keratin14 (KRT14) -promoter leading to deletion of Misu in all basal, undifferentiated cells of the epidermis (K14Misu^Δ/Δ^).

All mouse lines were bred to a mixed genetic background of CBA×C57BL/6J. Primers to identify Misu^wt^, Misu^flox^ and Misu^Δ^ alleles are F1 (5′CCC CCA CTG CTG CTC AAC G) and R1 (5′CAA TGC CAC CAC AAC CTC CTT).

### Hair cycle analyses

Total of 97 Misu −/− mice with their wild-type controls and 29 K14Misu^Δ/Δ^ with their Misu^f/f^ control mice were genotyped and grouped by gender. Samples were taken from same regions from dorsal skin and process for H&E staining. Each mouse was classified into specific stages of the hair cycle based on established morphological guidelines [51]. The total numbers were represented as percentages.

### tRNA bisulfite sequencing

Bisulfite conversion of tRNA was carried out as previously described [21]. cDNA was PCR amplified using primers specific for the deaminated sequences of tRNA-998^Leu (CAA)^ and tRNA-1911^Leu (CAA)^. tRNA^Leu (CAA)^ sequences were obtained from the Genomic tRNA Database (http://gtrnadb.ucsc.edu/). Primer sequences are as follows: Fw_Leu_De: 5′GAT GGT TGA GTG GTT TAA GGT GTT, Rv_Leu998_De: 5′CAC CTC CAA AAA AAA CCA AAA C and Rv_Leu1911_De: 5′CAC CTC CAT TCA AAA ACC AAA AC.

### BrdU labelling

DNA label-retaining cells (LRC) were generated by repeated BrdU (Sigma) injections of neonatal mice at P10 [25]. For LRC assays animals were chased for the times indicated. For tracing migration of bulge LRC into the hair germ, animals were sacrificed at P47 [4]. LRC were detected by BrdU immunostaining in tail skin whole mounts. Z-stack volumes of random areas of the slide were collected using a confocal microscope (Leica SP5). Maximum projected images were quantified using Volocity software (PerkinElmer). Images were segmented to identify and measure intensity of BrdU-positive cells constrained to specific Regions Of Interest (ROI) defining the whole hair follicle (bulge and hair germ), high and low bulge region and hair germ. Frequency distributions of BrdU intensity were calculated with Microsoft Office Excel 2007 software (Microsoft).

### Tissue staining

Immunostainings were performed on 10 µm paraffin sections. After citrate epitope retrieval, sections were permeabilized for 5 minutes with 0.2% Triton×100 at room temperature, blocked for 1 hour with 5% FCS and incubated overnight with the appropriate antibody dilution. Immunostaining on cryosections or cultured cells was performed as for paraffin after fixation for 10 minutes in 4% paraformaldehyde at room temperature. Tail epidermal whole mounts were prepared and immunolabelled as described previously [25], [36].

For LacZ staining, whole mounts of embryos or freshly obtained skin samples were fixed for 30 minutes at room temperature in buffer containing 0.1 M phosphate buffer, 5 mM EGTA, 2 mM MgCl2 and 0.2% glutaraldehyde. Samples were then washed three times for 15 minutes each in wash buffer (2 mM MgCl2 and 0.1% Nonidet P40 in 0.1 M phosphate buffer) and stained for 12 hours in a solution consisting of 1 mg/ml X-gal (Melford), 5 mM K_3_Fe(CN)_6_ and 5 mM K_4_Fe(CN)_6_ in wash buffer. The skin samples were then embedded in paraffin, sectioned at 10 µm and stained with eosin or used for stainings.

Primary antibodies were used at the following dilutions: rabbit monoclonal antibody to Ki67 (1∶100; SP6, Vector Labs), rabbit polyclonal anti mouse keratin 14 (1∶2000; Covance), rabbit polyclonal anti mouse keratin 10 (1∶500; Covance), mouse monoclonal anti Gata3 (1∶50; HG3-31, Santa Cruz Biotechnology), rabbit polyclonal anti Lef1 (1∶50; Cell Signaling Technology), mouse polyclonal anti Dlx3 (1∶200; Abnova), guinea pig polyclonal anti hair keratins 31, 71 and 72 (1∶200; Progen), rabbit polyclonal anti keratin 6 (1∶5.000; Babco), rabbit polyclonal to phosphor-Smad1/5/8 (1∶50; Cell Signaling Technology), rabbit polyclonal CUK-1079-A antibody to mouse Misu (1∶1000; produced by Covalab), mouse monoclonal to keratin 15 (1∶1000) [25]), rat monoclonal anti BrdU (1∶100; Abcam), goat polyclonal anti P-cadherin (1∶100; R & D Systems), rat monoclonal anti α6 integrin (1∶500; GoH3, AbD Serotec), Secondary antibodies (Alexa Fluor 594- and 488-conjugated anti-rabbit, mouse, rat and guinea pig, Invitrogen) were added at a dilution of 1∶500 for 1 hour at room temperature together with DAPI to label nuclei.

White field images were acquired using an Olympus IX80 microscope and a DP50 camera. Confocal images were acquired on a Leica TCS SP5 confocal microscope. Z-stacks were acquired at 100 Hz with an optimal stack distance and 1024×1024 dpi resolutions. Z-stack projections were generated using the LAS AF software package (Leica Microsystems). All the images were processed with Photoshop CS4 (Adobe) software.

### Protein extraction and Western blotting

Proteins were extracted from cultured keratinocytes or total skin. 1 cm^2^ pieces of total back skin (dermis and epidermis) were snap-frozen in liquid N2, transferred to lysis buffer (1% NP-40, 200 mM NaCl, 25 mM Tris-HCl, pH 8, 1 mM DTT) including protease inhibitor cocktail (Roche) and homogenised for 30 seconds. Samples were incubated on ice for 20 minutes. Protein lysates were cleared by centrifugation at 13,000 rpm. Total protein concentration was quantified using Dc Protein Assay (Bio-Rad). Equal amounts of protein were run in 7.5% polyacrylamide gels and blotted onto Hybond-P PVDF membranes (GE Healthcare), which were incubated in TBST-blocking solution (Tris-buffered saline, pH 8.8, with 5% skimmed milk powder). Blots were incubated overnight at 4°C with primary antibodies, washed and incubated with the appropriate HRP-conjugated secondary antibodies (GE Healthcare). α-Tubulin (Sigma) was used as a loading control. The chemiluminescent signal was detected using the ECL Plus Detection System (GE Healthcare).

### RNA extraction and quantitative RT–PCR (QPCR)

Total RNA from mouse skin or cultured keratinocytes was prepared using Trizol reagent (Invitrogen) according to the manufacturer's instructions. Total RNA from flow-sorted cells was purified using Pure-Link RNA Micro Isolation Kit (Invitrogen). Double-stranded cDNA was generated from 1 µg total RNA using Superscript III First-Strand Synthesis kit (Invitrogen) and random hexamer primers (Promega). A minimum of two independent biological and three technical replicates was analysed. In case of Itgá6/CD34^+ve^-sorted cells one sample was pooled from four mice. For FDG-sorted cells one sample was pooled from one mouse.

Real-time PCR amplification and analysis was conducted using the 7900HT Real-Time PCR System (Applied Biosystems). The standard amplification protocol was used with pre-designed probe sets and TaqMan Fast Universal PCR Master Mix (2×) (Applied Biosystems). Probe set Mm00520224_m1 and Mm00487803_m1 were used to amplify mouse NSun2 (Misu) and c-Myc from total skin. The following probes were used to amplify selected genes from flow-sorted cells: α6 integrin (Mm01333831_m1), FGF-18 (Mm00433286_m1), CD34 (Mm00519283_m1), NFATc1 (Mm00479445_m1), Sox9 (Mm00448840_m1), Lgr5 (Mm00438890_m1), Sox6 (Mm00488393_m1), Wnt5a (Mm00437347_m1), Lef1 (Mm00550265_m1), Gata3 (Mm01337569_m1) and Keratin 72 (Mm00495207_m1). GAPDH expression (4352932E) was used to normalize samples using the ΔCt method.

### Cloning, cell culture, transfection, and reporter assays

The −2068 to −48 bp DNA fragment of the mouse Misu promoter was cloned into the pGL3-Basic vector (Promega). Plasmids for the reporter assays included pCDNA 3.1-hLef1-V5 (Lef1), pCDNA 3.1-ΔN63-hLef1-V5 (ΔLef1), and pCDNA 3.1-S33Y mCTNNB1 (β-catenin). pLef7-fos-luc (pLef7) was kindly provided by R. Grosschedl [52]. Hela cells were grown in DMEM (Invitrogen) supplemented with 10% fetal calf serum (FCS) in a humidified atmosphere at 37°C and 5% CO2. Cells were transiently co-transfected with the promoter construct, pRL-TK renilla as an internal control and the indicated plasmids using Lipofectamine LTX transfection reagent (Invitrogen). After recovery, cells were grown in media containing 0.2%FCS. Luciferase activity was measured 36–48 hours after the transfection using the Dual-Luciferase Reporter Assay System (Promega) on Glomax (Promega). Each transfection was carried out in triplicate and the experiment was repeated twice.

### Flow cytometry, cell sorting, and cell cycle analysis

To isolate mouse keratinocytes from dorsal back skin we rinsed mouse back skin in 10% Betadine and 70% ethanol and washed it in PBS. The dermal side was thoroughly scraped to remove excess fat. The tissue was then floated on 0.25% Trypsin without EDTA (Invitrogen) for 2 hours at 37°C or overnight at 4°C. The epidermis was subsequently scraped from the dermis, minced using scalpels, disaggregated by gentle pipetting and filtered through a 70 ìm cell strainer. Trypsin was inactivated by addition of low-calcium medium with 10% FCS. The cells were pelleted and resuspended in the following antibodies: PE-conjugated Itgá6 (clone GoH3, eBiosciences), Alexa Fluor 647-conjugated CD34 (RAM34, eBiosciences) and goat polyclonal anti-P-cadherin (R & D Systems). After incubation for 45 minutes at 4°C, cells were washed twice in PBS. For detection of P-cadherin, cells were incubated for 10 minutes at 4°C with anti-goat Alexa Fluor 647-congugated secondary antibody (Invitrogen).

Cells were gated using forward versus side scatter to eliminate debris. Doublet discrimination was carried out using pulse width. The viable cells were then gated by their exclusion of DAPI using a 450/65 nm filter. Itgá6 PE stained cells were detected using a 580/30 nm filter and Alexa 647 cells were detected using a 670/30 nm filter. Cells were sorted with a MoFlo high-speed sorter (Beckman Coulter).

For cell cycle analysis of Itgá6^high^/CD34^+ve^ or Itgá6^low^/P-cadherin^high^ cell populations, cells were fixed with 1% paraformaldehyde for 5 minutes after immunolabelling, transferred to cold 70% ethanol and incubated for at least 1 hour before stained with propidium iodide (PI) or DAPI (Sigma). After incubating the cells for 1 hour in RNase, analysis was carried out on a CyAN ADP analyzer (Beckman Coulter).

For detection of intracellular β-galactosidase activity, mouse keratinocytes from mice in early anagen (P21) were loaded with fluorescein-di-β-D-galactopyranoside (FDG) (Sigma) using hypotonic shock. Briefly, equal volume of cells was mixed with warm 2× hypothonic shock solution (2 mM FDG in water) and incubated for 30 seconds at 37°C, then cold media was added. Cells were washed and subsequently stained with the appropriate antibodies. Cells were then sorted based on FDG (fluorescence detected using a 530/40 nm filter), after gating out dead cells (based on DAPI staining).

### Cell culture of mouse keratinocytes, clonal growth, and differentiation assay

Mouse keratinocytes were isolated as described above and cultured on mitomycin-treated J2-3T3 feeder cells on collagen type I (BD Biosciences) coated plates (BD Falcon). Mouse keratinocytes were grown in low-calcium FAD media (one part Ham's F12, three parts Dulbecco's modified Eagle's medium, 18 mM adenine and 0.05 mM calcium) supplemented with 10% FCS and a cocktail of 0.5 µg/ml of hydrocortisone, 5 µg/ml insulin, 10^−10^ M cholera enterotoxin, and 10 ng/ml epidermal growth factor (HICE cocktail) and maintained in a humidified atmosphere at 32°C and 8% CO_2_.

Clonal growth was assayed by culturing 500 to 2500 Itgá6^high^/CD34^+ve^ sorted cells or 5000 to 10000 viable epidermal cells per well in 6-well plates for 3 weeks. Cells were fixed and stained with 1% Rhodamine B. Three independent experiments were conducted.

### Chromatin immunoprecipitation (ChIP)

Mouse keratinocytes were isolated from mouse skin in anagen and processed according Chromatin immunoprecipitation Assay Kit (Upstate). Chromatin was incubated with control anti-rabbit IgG and anti-Lef1 (Cell Signaling) antibody overnight at 4°C. The samples were eluted after washing. PCR reactions were performed by sets of specific primers: Misu TCT GTG CGG TCC TTT CTA CC (forward) and CGC GTC CTG CTA GCT ATG TT (reverse); Msx2 AAG GGA GAA AGG GTA GAG (forward) and CCC GCC TGA GAA TGT TGG (reverse) and GAPDH TAC TAG CGG TTT TAC GGG CG (forward) and TCG AAC AGG AGG AGC AGA GAG CGA (reverse).

### Statistical analysis

The significance of quantitative data was tested using the unpaired, two-tailed Student's T test.

## Supporting Information

Figure S1 Validation of Misu −/− mice. (A) Exon-intron organization of murine MISU gene, showing the SUN domain (red box) and the Gene Trap insertion (filled blue box). (B) Gene-specific PCR to detect MISU (upper panel) and LacZ alleles (lower panel). (C) QPCR using total back skin shows lack of Misu-mRNA in Misu −/− mice. (D) Western blot confirms deletion of Misu-protein. Tubulin (Tub) was used as loading control.
(TIF)

Figure S2 Misu is expressed in committed progenitor cells. (A–H) Immunohistochemistry of LacZ (blue) of hair follicles of adult mice in anagen at P31 co-stained for the proliferation marker Ki67 (A), markers for the cortex and pre-cortex; Dlx3 (B) and keratin 31 (C), markers for the inner root sheath; Gata3 (D) and keratin 71 and 72 (E,F), a marker for the companion layer; keratin 6 (G) and a marker for the outer root sheath; keratin 14 (H). Scale bars: 50 µm.
(TIF)

Figure S3 LacZ-expression and Misu protein are absent in controls. (A,B) LacZ staining of wild-type skin at telogen (A) and anagen (B). Arrows indicate unspecific staining in sebaceous glands. Sections are counterstained with Eosin. (C) Confocal image of Misu −/− hair follicle in anagen co-stained for K15 (red), Misu (green) and nulei (DAPI, in blue). Scale bars: 50 µm.
(TIF)

Figure S4 Expression of bulge and hair germ markers. (A–C) Whole mount labelling for Itgα6 (red), P-cadherin (P-cadh) (green) and DAPI (blue) in tail skin. Scale bar: 100 µm.
(TIF)

Figure S5 Expression profile of FDG^+ve^ cells at the onset of anagen. (A–B) Gating for FDG^+ve^ and FDG^−ve^ epidermal cells from Misu +/− mice at P21 using flow cytometry. Cells were gated by F2Log (autofluorescence) versus F1Log (FDG). FDG^+ve^ cells were sorted as indicated by the blue square and FDG^−ve^ cells were sorted as indicated by the black square. Epidermal cells from wild-type mice are negative for FDG (A). (C–G) QPCR for the indicated genes of total RNA isolated from FDG^+ve^ and FDG^−ve^ sorted epidermal cells. Error bars indicate SEM (n = 3).
(TIF)

Figure S6 Cell cycle analysis of stem and progenitor cells using flow cytometry. (A–C) Gating of epidermal cells using flow cytometry to analyse the cell cycle profile of bulge stem cells (A), hair germ cells (B) at P21, and bulge stem cells at P24 (C).
(TIF)

Figure S7 Flow cytometry analysis for bulge stem cells and progenitor cells in anagen and telogen. (A) Flow cytometry analysis for expression of Itgα6 and CD34 in epidermis at anagen (P30) and (B) Itgα6 and P-Cadherin at telogen (P49) in wild-type and Misu −/− mice.
(TIF)

Figure S8 Conditional deletion of Misu in skin increases dormancy of bulge stem cells. (A) Schematic overview of MISU^f/f^ allele. Exon-intron organization of murine MISU gene, showing the SUN domain (red box) and LoxP sites flanking exon 6 (blue triangles). (B–C) Validation of conditional deletion of Misu in the epidermis in K14Misu^Δ/Δ^ mice. QPCR using total back skin shows a decrease of Misu-mRNA levels in K14Misu^Δ/Δ^ mice, especially in anagen (P27), when Misu-mRNA levels are the highest in the control Misu^f/f^ mice (B). QPCR using total back skin and epidermis of mice in telogen (P49) shows a specific reduction in Misu-mRNA levels in K14Misu^Δ/Δ^ epidermis compared to total skin (C). (D) Epidermis in telogen (P49) analysed by flow cytometry for expression of Itgα6 and CD34. The percentage of Itgα6^low^/CD34^+ve^ and Itgα6^high^/CD34^+ve^ cells is shown ± SEM (n = 3). The red bars in (D) indicate a cell population enriched in K14Misu^Δ/Δ^ epidermis. The grey box indicates cells sorted as population L (Itgα6^low^/CD34^+ve^) and the black box cells sorted as population H (Itgα6^high^/CD34^+ve^). (E) QPCR for the sorted cell populations indicated in (D) from K14Misu^Δ/Δ^ and Misu^f/f^ mice. RNA levels were normalized to GAPDH and values for K14Misu^Δ/Δ^ versus controls (Misu^f/f^) measured. Error bars represent SEM (n = 4).
(TIF)

Figure S9 Lack of Misu impairs migration of LRC from the bulge into the hair germ. (A,B) BrdU (green) incorporation into wild-type (A) and Misu −/− (B) epidermis 2 hours after last BrdU injection. Nuclei are counterstained with DAPI. (C–F) Frequency distributions of intensity of the BrdU-label in the whole hair follicle (bulge plus hair germ) (C), the high bulge (D), the low bulge (E) and the hair germ (F). Error bars indicate SEM (n = 5 hair follicles per mouse, from 4 wt and 4 Misu −/− mice). Scale bar: 100 µm in (A,B).
(TIF)

Figure S10 Entry into anagen is delayed in Misu −/− and K14Misu^Δ/Δ^ mice. (A) Percentage of Misu −/− females and their control littermates represented in Table S1. (B–G) Haematoxylin and Eosin (H&E) staining (B,C) and immunohistochemistry for Ki67 (D,E) of dorsal skin sections of Misu −/− and wild-type hair follicles at P19 (B,C), and P27 (D,E). (F–K) Histology of dorsal skin sections at indicated postnatal days shows delay of entry into anagen in K14Misu^Δ/Δ^ mice compared to controls (Misu^f/f^). Immunohistochemistry for Ki67 of skin sections at P21 (F,G). Haematoxylin and Eosin staining of skin sections at indicated postnatal days (H–K). (L–S) Misu −/− hair follicles are indistinguishable from their wild-type controls at later stages in anagen at P31. Confocal images of wid-type and Misu −/− hair follicles stained for Lef1 (green) (L,M), hair keratin 72 (green) and Dlx3 (red) (N,O), phospho-Smad1/3/5 (green) (P,Q) and hair keratin 31 (green) and Gata3 (red) (R,S). Nuclei are counterstained with Haematoxylin (B–K) and DAPI (L–S). Scale bar: 200 µm (B–E); 100 µm (F–S).
(TIF)

Table S1 Hair cycle staging of Misu −/− mice and their control littermates. For each postnatal day (P), mice are grouped by genotype and gender. The total number of mice is indicated with N. Each mouse is classified into specific hair cycle phases based on established morphological guidelines. Anagen I–IIIa is defined as period starting from the onset of mitotic activity in the hair germ (I) to cells show differentiation into all follicular components (IIIa). Anagen IIIb–VI includes the stage of melanocyte activation (IIIb) to a new hair shaft emerges from skin surface (VI).
(DOCX)
