# Supplementary figures and images for: The RNA–Methyltransferase Misu (NSun2) Poises Epidermal Stem Cells to Differentiate
Source: PLoS Genet. 2011 Dec 1;7(12):e1002403. doi: 10.1371/journal.pgen.1002403 (PMC3228827; doi:10.1371/journal.pgen.1002403)

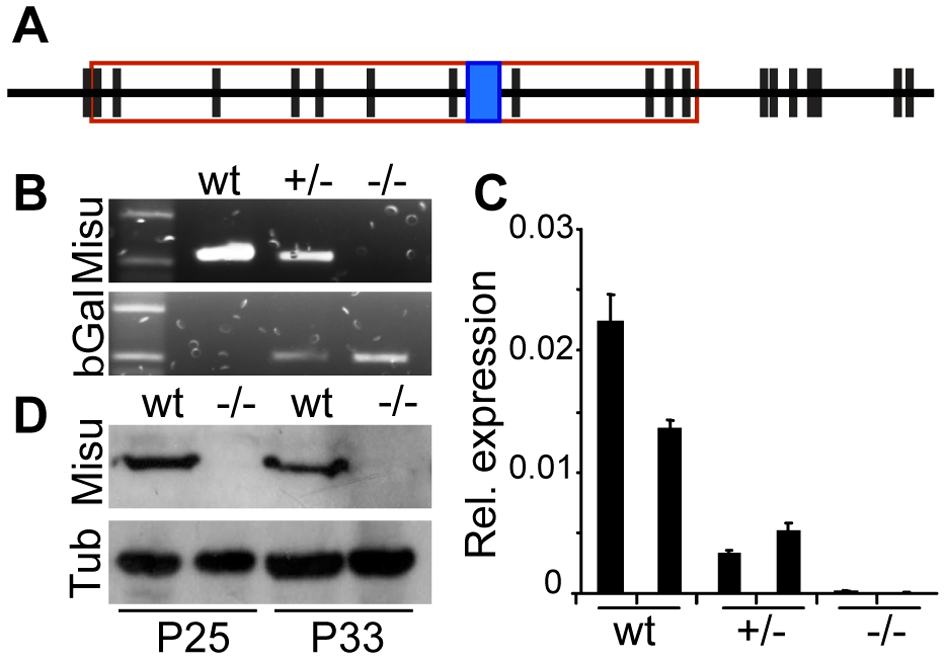

Supplement: Figure S1 — Validation of Misu −/− mice. (A) Exon-intron organization of murine MISU gene, showing the SUN domain (red box) and the Gene Trap insertion (filled blue box). (B) Gene-specific PCR to detect MISU (upper panel) and LacZ alleles (lower panel). (C) QPCR using total back skin shows lack of Misu-mRNA in Misu −/− mice. (D) Western blot confirms deletion of Misu-protein. Tubulin (Tub) was used as loading control. (TIF) [file pgen.1002403.s001.tif]

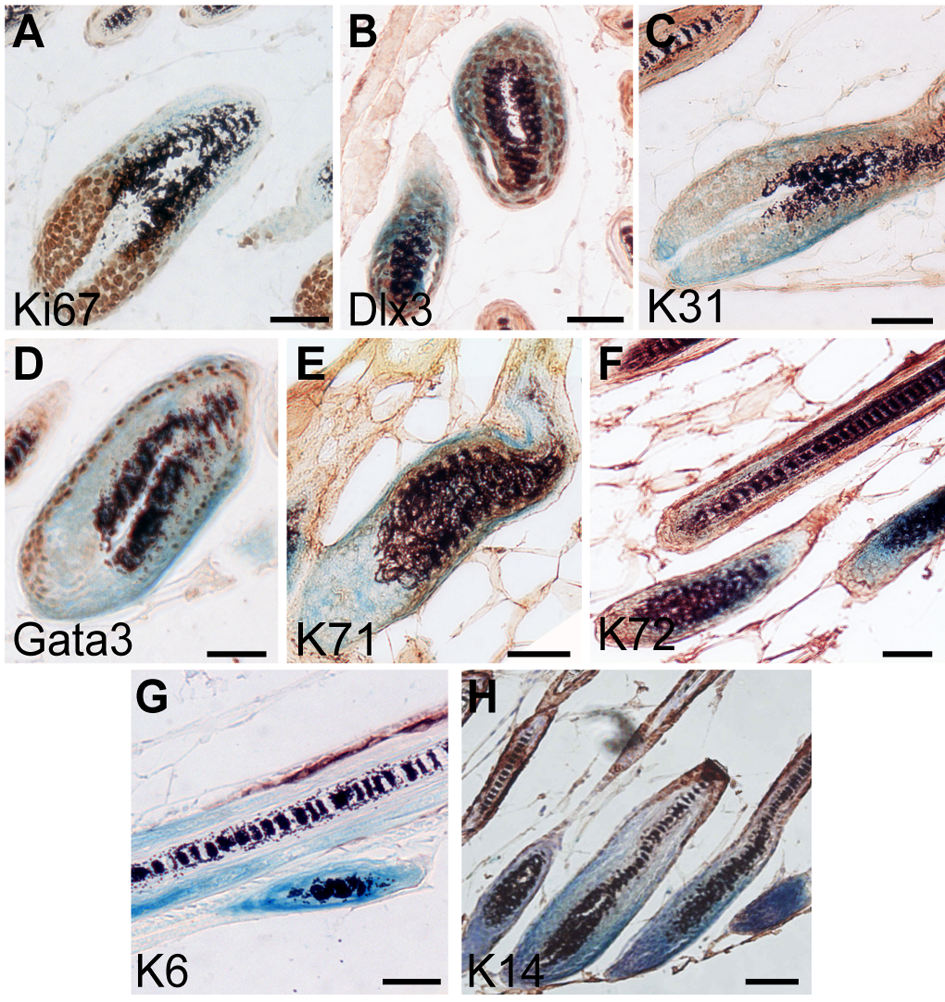

Supplement: Figure S2 — Misu is expressed in committed progenitor cells. (A–H) Immunohistochemistry of LacZ (blue) of hair follicles of adult mice in anagen at P31 co-stained for the proliferation marker Ki67 (A), markers for the cortex and pre-cortex; Dlx3 (B) and keratin 31 (C), markers for the inner root sheath; Gata3 (D) and keratin 71 and 72 (E,F), a marker for the companion layer; keratin 6 (G) and a marker for the outer root sheath; keratin 14 (H). Scale bars: 50 µm. (TIF) [file pgen.1002403.s002.tif]

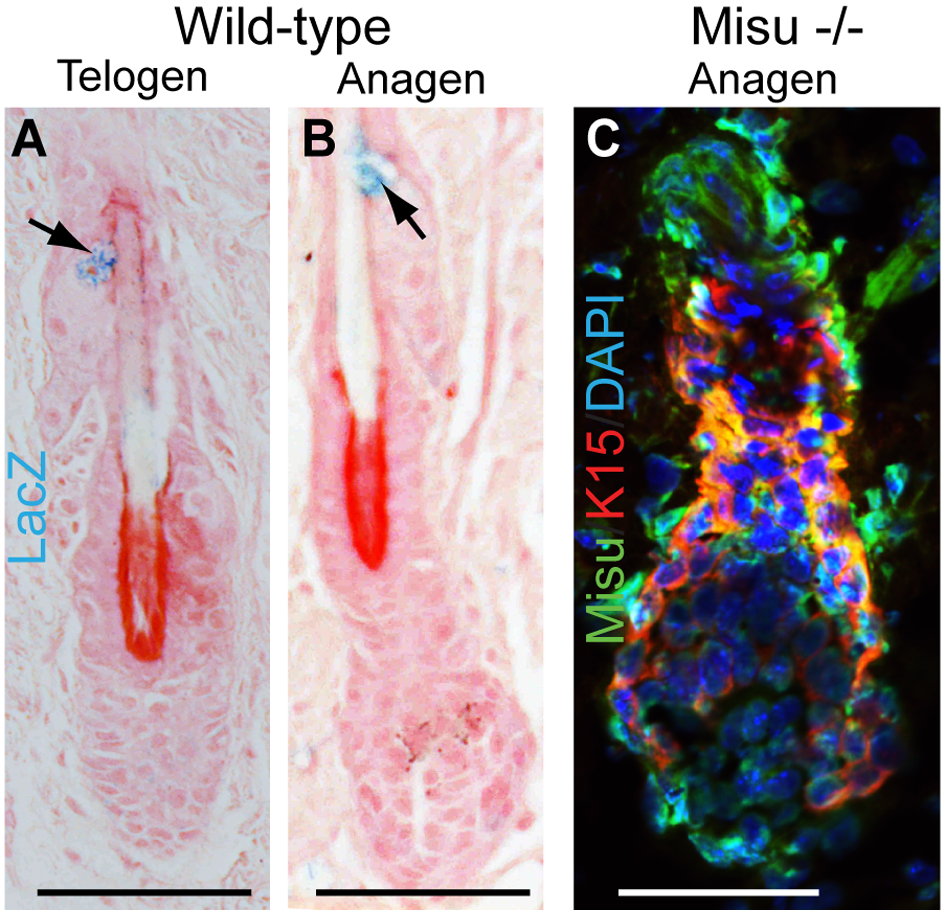

Supplement: Figure S3 — LacZ-expression and Misu protein are absent in controls. (A,B) LacZ staining of wild-type skin at telogen (A) and anagen (B). Arrows indicate unspecific staining in sebaceous glands. Sections are counterstained with Eosin. (C) Confocal image of Misu −/− hair follicle in anagen co-stained for K15 (red), Misu (green) and nulei (DAPI, in blue). Scale bars: 50 µm. (TIF) [file pgen.1002403.s003.tif]

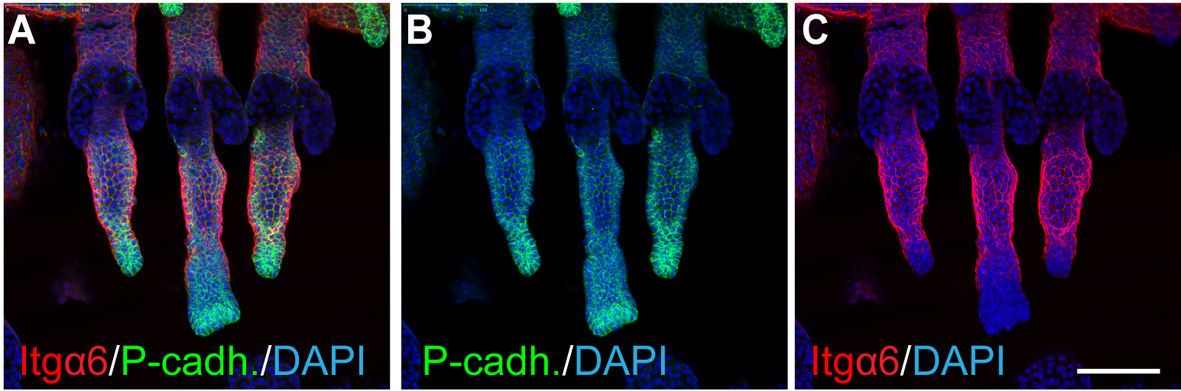

Supplement: Figure S4 — Expression of bulge and hair germ markers. (A–C) Whole mount labelling for Itgα6 (red), P-cadherin (P-cadh) (green) and DAPI (blue) in tail skin. Scale bar: 100 µm. (TIF) [file pgen.1002403.s004.tif]

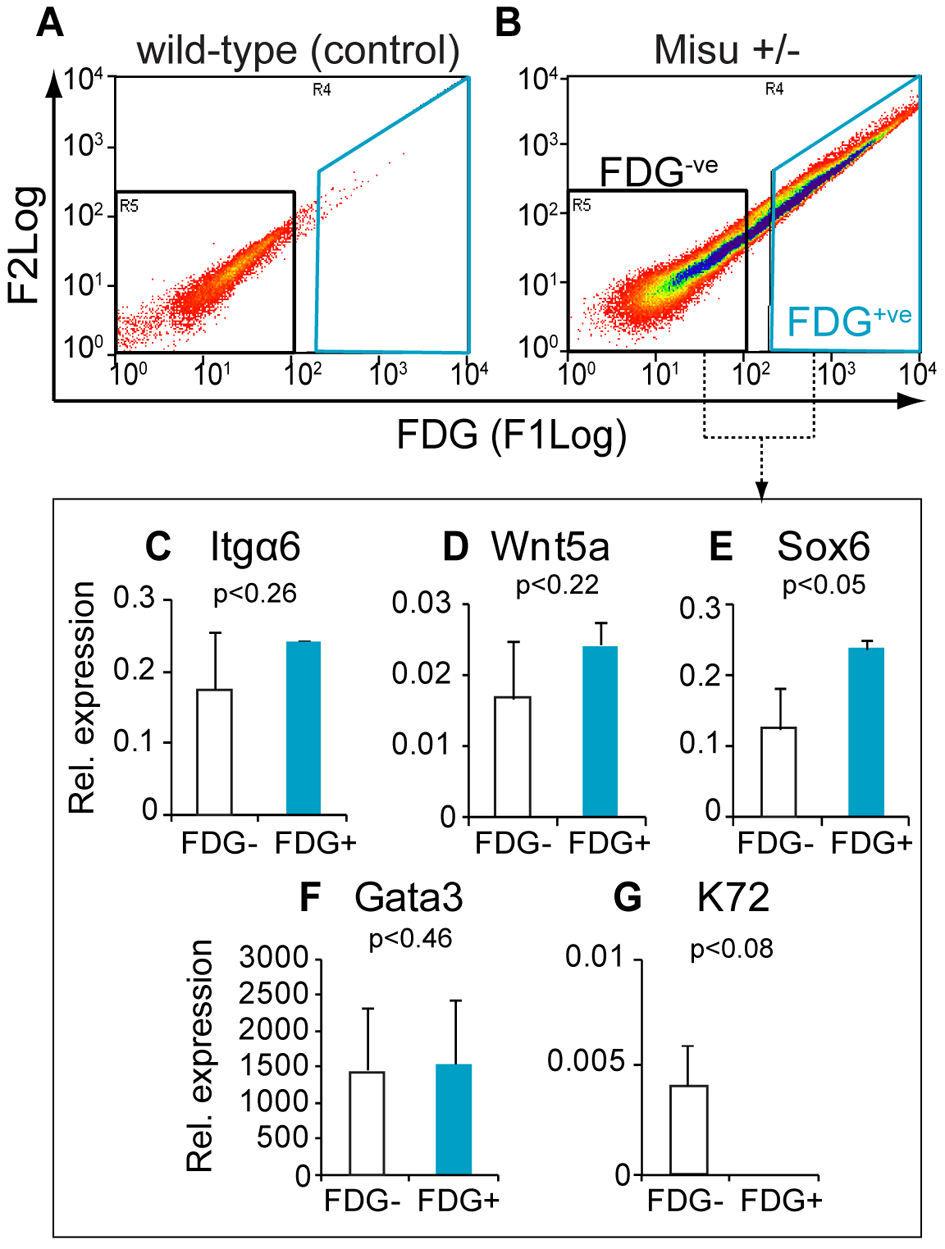

Supplement: Figure S5 — Expression profile of FDG+ve cells at the onset of anagen. (A–B) Gating for FDG+ve and FDG−ve epidermal cells from Misu +/− mice at P21 using flow cytometry. Cells were gated by F2Log (autofluorescence) versus F1Log (FDG). FDG+ve cells were sorted as indicated by the blue square and FDG−ve cells were sorted as indicated by the black square. Epidermal cells from wild-type mice are negative for FDG (A). (C–G) QPCR for the indicated genes of total RNA isolated from FDG+ve and FDG−ve sorted epidermal cells. Error bars indicate SEM (n = 3). (TIF) [file pgen.1002403.s005.tif]

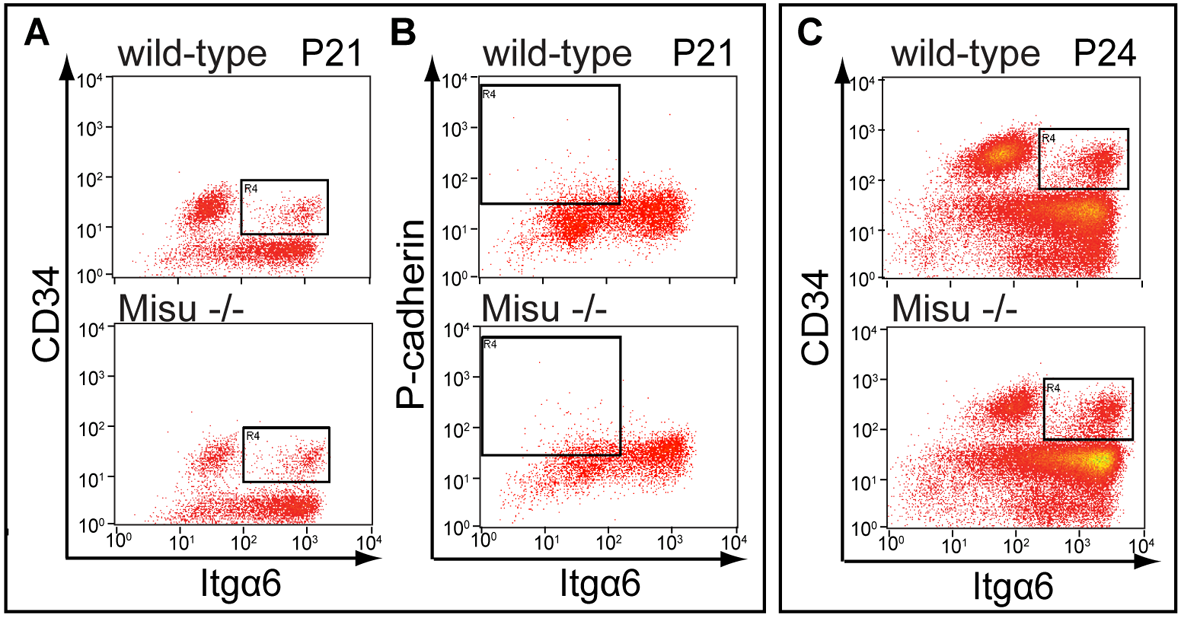

Supplement: Figure S6 — Cell cycle analysis of stem and progenitor cells using flow cytometry. (A–C) Gating of epidermal cells using flow cytometry to analyse the cell cycle profile of bulge stem cells (A), hair germ cells (B) at P21, and bulge stem cells at P24 (C). (TIF) [file pgen.1002403.s006.tif]

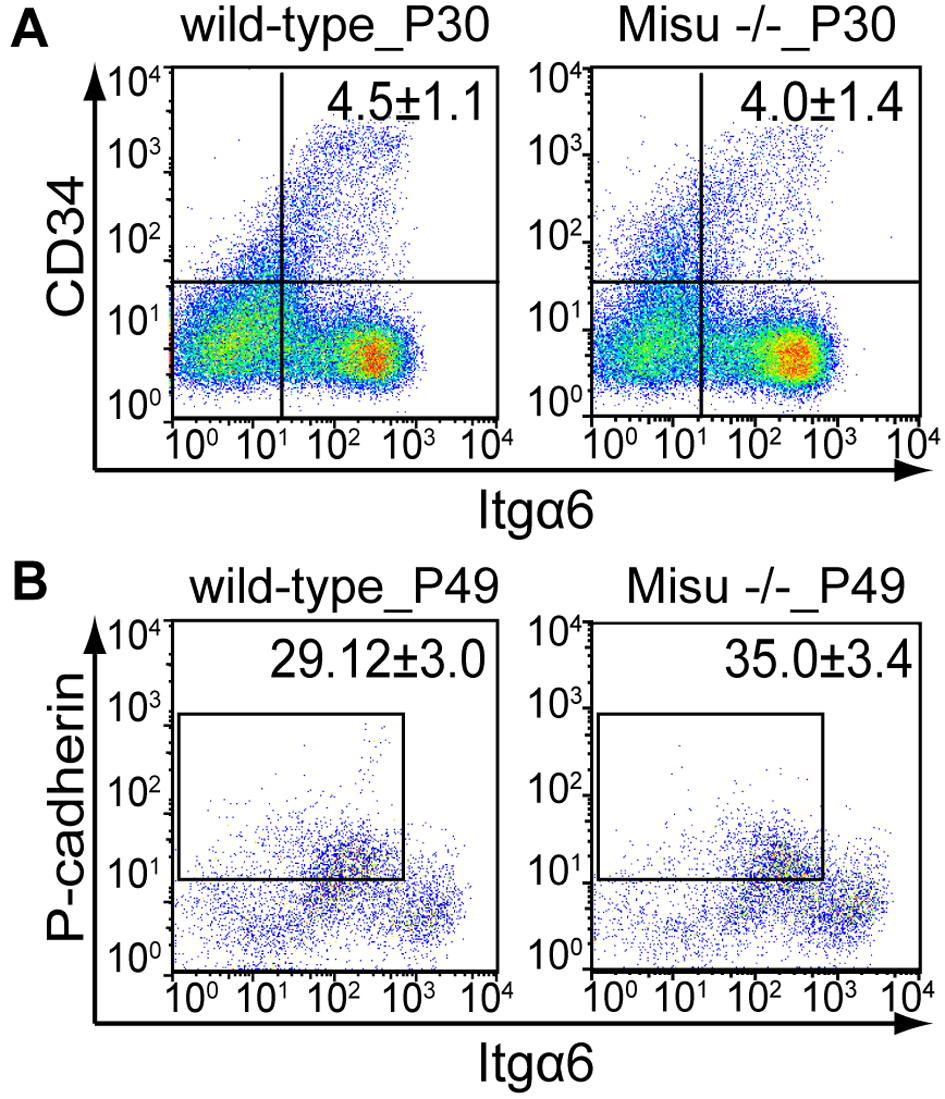

Supplement: Figure S7 — Flow cytometry analysis for bulge stem cells and progenitor cells in anagen and telogen. (A) Flow cytometry analysis for expression of Itgα6 and CD34 in epidermis at anagen (P30) and (B) Itgα6 and P-Cadherin at telogen (P49) in wild-type and Misu −/− mice. (TIF) [file pgen.1002403.s007.tif]

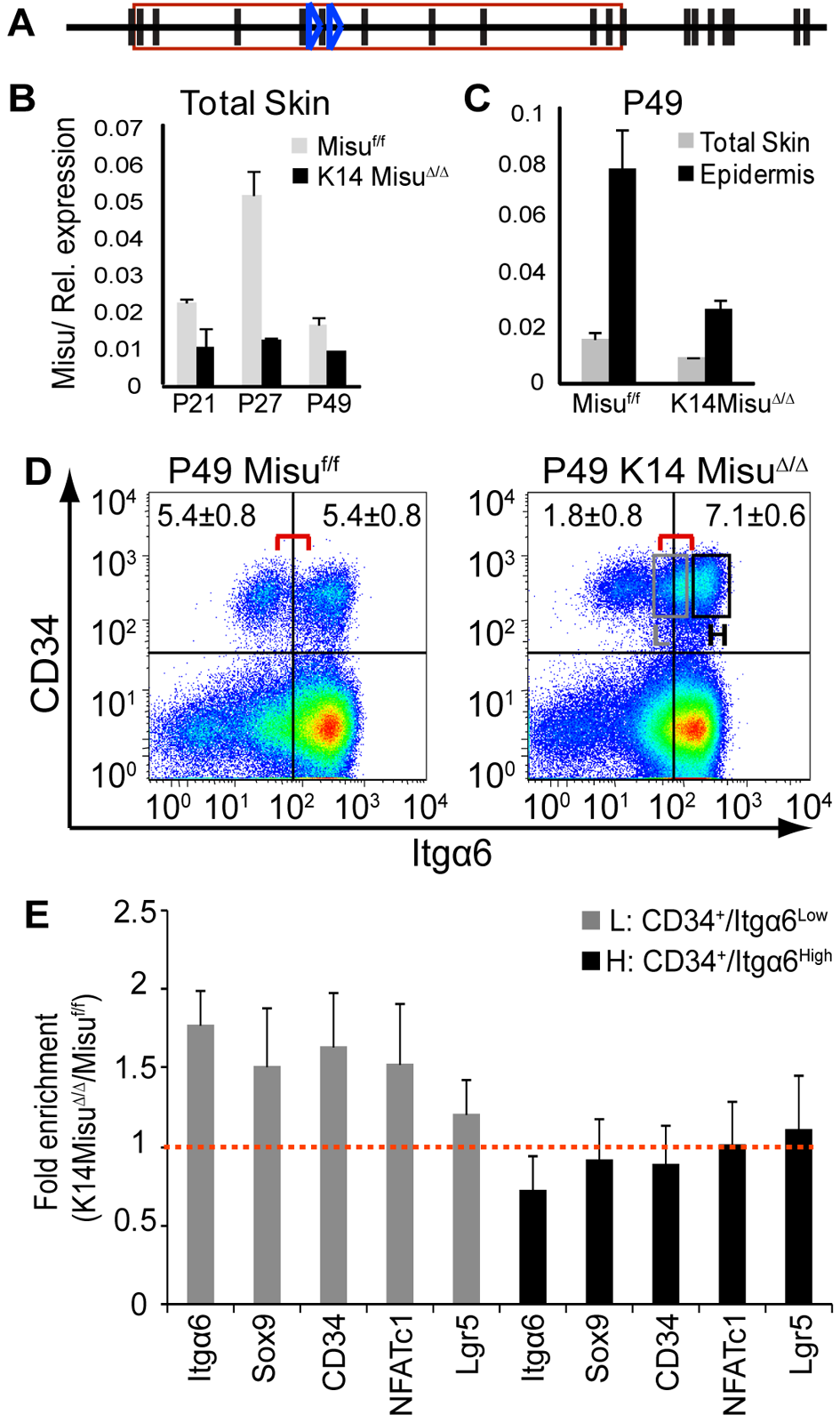

Supplement: Figure S8 — Conditional deletion of Misu in skin increases dormancy of bulge stem cells. (A) Schematic overview of MISUf/f allele. Exon-intron organization of murine MISU gene, showing the SUN domain (red box) and LoxP sites flanking exon 6 (blue triangles). (B–C) Validation of conditional deletion of Misu in the epidermis in K14MisuΔ/Δ mice. QPCR using total back skin shows a decrease of Misu-mRNA levels in K14MisuΔ/Δ mice, especially in anagen (P27), when Misu-mRNA levels are the highest in the control Misuf/f mice (B). QPCR using total back skin and epidermis of mice in telogen (P49) shows a specific reduction in Misu-mRNA levels in K14MisuΔ/Δ epidermis compared to total skin (C). (D) Epidermis in telogen (P49) analysed by flow cytometry for expression of Itgα6 and CD34. The percentage of Itgα6low/CD34+ve and Itgα6high/CD34+ve cells is shown ± SEM (n = 3). The red bars in (D) indicate a cell population enriched in K14MisuΔ/Δ epidermis. The grey box indicates cells sorted as population L (Itgα6low/CD34+ve) and the black box cells sorted as population H (Itgα6high/CD34+ve). (E) QPCR for the sorted cell populations indicated in (D) from K14MisuΔ/Δ and Misuf/f mice. RNA levels were normalized to GAPDH and values for K14MisuΔ/Δ versus controls (Misuf/f) measured. Error bars represent SEM (n = 4). (TIF) [file pgen.1002403.s008.tif]

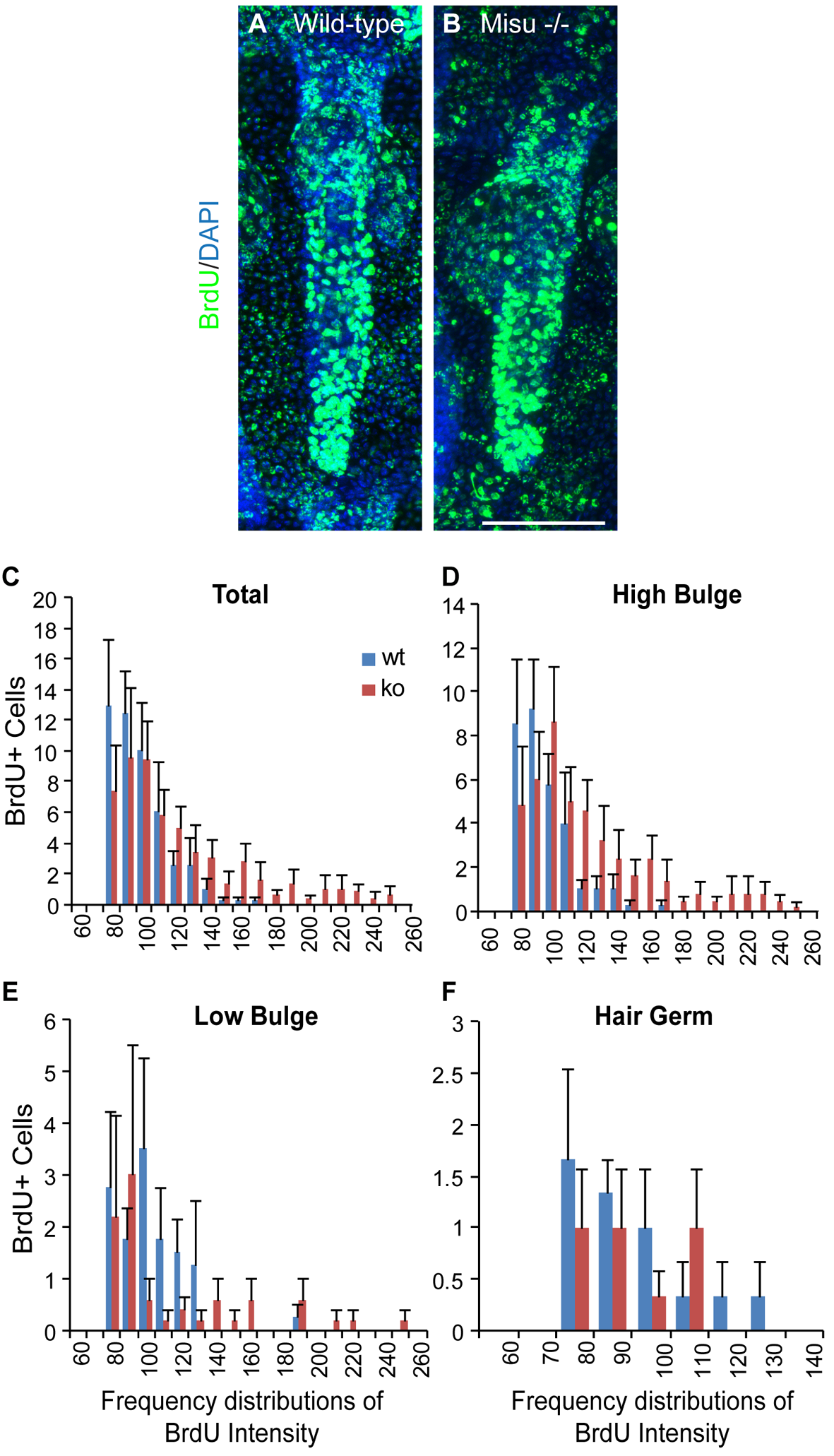

Supplement: Figure S9 — Lack of Misu impairs migration of LRC from the bulge into the hair germ. (A,B) BrdU (green) incorporation into wild-type (A) and Misu −/− (B) epidermis 2 hours after last BrdU injection. Nuclei are counterstained with DAPI. (C–F) Frequency distributions of intensity of the BrdU-label in the whole hair follicle (bulge plus hair germ) (C), the high bulge (D), the low bulge (E) and the hair germ (F). Error bars indicate SEM (n = 5 hair follicles per mouse, from 4 wt and 4 Misu −/− mice). Scale bar: 100 µm in (A,B). (TIF) [file pgen.1002403.s009.tif]

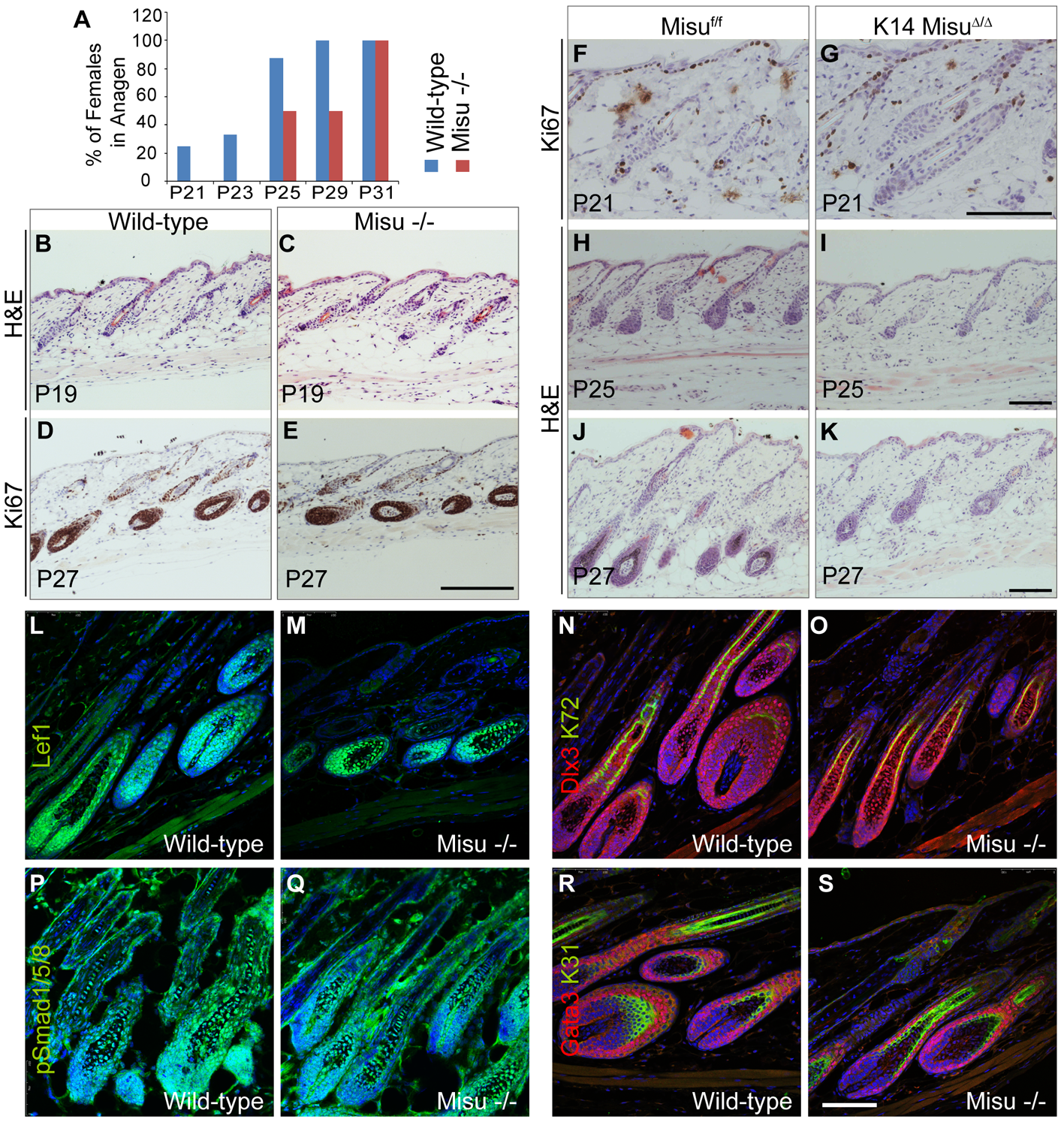

Supplement: Figure S10 — Entry into anagen is delayed in Misu −/− and K14MisuΔ/Δ mice. (A) Percentage of Misu −/− females and their control littermates represented in Table S1. (B–G) Haematoxylin and Eosin (H&E) staining (B,C) and immunohistochemistry for Ki67 (D,E) of dorsal skin sections of Misu −/− and wild-type hair follicles at P19 (B,C), and P27 (D,E). (F–K) Histology of dorsal skin sections at indicated postnatal days shows delay of entry into anagen in K14MisuΔ/Δ mice compared to controls (Misuf/f). Immunohistochemistry for Ki67 of skin sections at P21 (F,G). Haematoxylin and Eosin staining of skin sections at indicated postnatal days (H–K). (L–S) Misu −/− hair follicles are indistinguishable from their wild-type controls at later stages in anagen at P31. Confocal images of wid-type and Misu −/− hair follicles stained for Lef1 (green) (L,M), hair keratin 72 (green) and Dlx3 (red) (N,O), phospho-Smad1/3/5 (green) (P,Q) and hair keratin 31 (green) and Gata3 (red) (R,S). Nuclei are counterstained with Haematoxylin (B–K) and DAPI (L–S). Scale bar: 200 µm (B–E); 100 µm (F–S). (TIF) [file pgen.1002403.s010.tif]
