# Supplementary material for: The RNA–Methyltransferase Misu (NSun2) Poises Epidermal Stem Cells to Differentiate
Source: PLoS Genet. 2011 Dec 1;7(12):e1002403. doi: 10.1371/journal.pgen.1002403 (PMC3228827; doi:10.1371/journal.pgen.1002403)
Supplement: Table S1 — Hair cycle staging of Misu −/− mice and their control littermates. For each postnatal day (P), mice are grouped by genotype and gender. The total number of mice is indicated with N. Each mouse is classified into specific hair cycle phases based on established morphological guidelines. Anagen I–IIIa is defined as period starting from the onset of mitotic activity in the hair germ (I) to cells show differentiation into all follicular components (IIIa). Anagen IIIb–VI includes the stage of melanocyte activation (IIIb) to a new hair shaft emerges from skin surface (VI). (DOCX) [file pgen.1002403.s011.docx]

|  |  | **Males/1st post-natal Anagen** | | | |
| --- | --- | --- | --- | --- | --- |
|  | **Postnatal day** | **Total** | **Telogen** | **Anagen I-IIIa** | **Anagen IIIb-VI** |
| **Wild-type** | P21 | 5 |  | 5 |  |
|  | P23 | 5 |  | 1 | 4 |
|  | P25 | 5 |  | 1 | 4 |
|  | P29 | 3 |  |  | 3 |
|  | P31 | 1 |  |  | 1 |
| **Misu -/-** | P21 | 2 | 2 |  |  |
|  | P23 | 1 | 1 |  |  |
|  | P25 | 4 | 3 | 1 |  |
|  | P29 | 2 | 1 |  | 1 |
|  | P31 | 1 |  |  | 1 |
|  |  | **Females/1st post-natal Anagen** | | | |
| **Wild-type** | P21 | 6 | 4 | 2 |  |
|  | P23 | 6 | 4 | 2 |  |
|  | P25 | 5 | 1 | 3 | 1 |
|  | P29 | 5 |  | 2 | 3 |
|  | P31 | 1 |  |  | 1 |
| **Misu -/-** | P21 | 2 | 2 |  |  |
|  | P23 | 4 | 4 |  |  |
|  | P25 | 2 | 1 | 1 |  |
|  | P29 | 2 | 1 |  | 1 |
|  | P31 | 1 |  |  | 1 |
|  |  | **Males/2nd post-natal Anagen** | | | |
| **Wild-type** | P60 | 1 | 1 |  |  |
|  | P70 | 6 | 5 |  | 1 |
|  | P75 | 8 | 3 |  | 5 |
|  | P80 | 7 | 3 |  | 4 |
| **Misu -/-** | P60 | 2 | 2 |  |  |
|  | P70 | 3 | 3 |  |  |
|  | P75 | 4 | 4 |  |  |
|  | P80 | 3 | 3 |  |  |
